# Origin of the Springback Effect in Ambient-Pressure-Dried Silica Aerogels: The Effect of Surface Silylation

**DOI:** 10.3390/gels9020160

**Published:** 2023-02-16

**Authors:** Fabian Zemke, Julien Gonthier, Ernesto Scoppola, Ulla Simon, Maged F. Bekheet, Wolfgang Wagermaier, Aleksander Gurlo

**Affiliations:** 1 Chair of Advanced Ceramic Materials, Institute of Materials Science and Technology, Faculty III Process Sciences, Technische Universität Berlin, Straße des 17. Juni 135, 10623 Berlin, Germany; 2 Department of Biomaterials, Max Planck Institute of Colloids and Interfaces, Am Mühlenberg 1, 14476 Potsdam, Germany

**Keywords:** aerogel, xerogel, springback effect, silica, silylation, ambient pressure drying, trimethylchlorosilane, triethylchlorosilane, hexamethyldisilazane

## Abstract

Ambient pressure drying (APD) can prospectively reduce the costs of aerogel fabrication and processing. APD relies solely on preventing shrinkage or making it reversible. The latter, i.e., the aerogel re-expansion after drying (so-called springback effect—SBE), needs to be controlled for reproducible aerogel fabrication by APD. This can be achieved by an appropriate surface functionalization of aerogel materials (e.g., SiO_2_). This work addresses the fabrication of monolithic SiO_2_ aerogels and xerogels by APD. The effect of several silylation agents, i.e., trimethylchlorosilane, triethylchlorosilane, and hexamethyldisilazane on the SBE is studied in detail, applying several complementary experimental techniques, allowing the evaluation of the macroscopic and microscopic morphology as well as the composition of SiO_2_ aerogels. Here, we show that some physical properties, e.g., the bulk density, the macroscopic structure, and pore sizes/volumes, were significantly affected by the re-expansion. However, silylation did not necessarily lead to full re-expansion. Therefore, similarities in the molecular composition could not be equated to similarities in the SBE. The influences of steric hindrance and reactivity are discussed. The impact of silylation is crucial in tailoring the SBE and, as a result, the APD of monolithic aerogels.

## 1. Introduction

Aerogels have proven to be promising materials for various applications, e.g., thermal insulation, drug delivery, catalysis, oil spill absorbents, and Cherenkov detectors [1]. In particular, their low thermal conductivity, low density, large specific surface area, and very high porosity give rise to these application fields [2]. Aerogels obtained by a sol-gel process undergo an insignificant change in their physical properties while drying [3].

Silica aerogels are one of the most studied systems and thus have sparked interest in understanding the influence of synthesis parameters on the material structure and properties [4,5,6]. Silica aerogels are synthesized either from sodium silicate or alkoxides, e.g., tetraethyl-orthosilicate (TEOS) [7]. During the initial steps, hydrolysis and condensation reactions take place and the system undergoes a sol-to-gel transformation. Water can react with the alkoxide precursor to form either completely hydrolyzed silicic acid or a partially hydrolyzed product with one or more silanol or ethoxy end groups, splitting off ethanol in the process. The latter can then either react with the alkoxide to form an interconnected network while producing ethanol, or two silanol end groups condensate, splitting off water. During these first steps, primary particles are formed that build the backbone of the structure [8,9]. All of these reactions strongly depend on the system’s pH value and determine the gel network’s interconnectivity [10].

Drying is the most critical processing step, as capillary forces arising from the evaporation of the liquid inside the porous gel structure lead to shrinkage of the material. While supercritical drying (SCD) remains the standard for the production of aerogels [1], other approaches, such as ambient pressure drying (APD), are also feasible [4]. On the other hand, without adequate preparation, evaporative drying with its considerable shrinkage and changes in porosity results in xerogels. Whereas SCD bypasses the liquid/vapor phase boundary due to supercritical conditions, APD often relies on a surface modification of the gel network [11,12,13,14]. Nonetheless, the gel structure must withstand the induced compressive stresses. The capillary pressure is influenced by the pore size and the surface tension of the liquid, as well as the contact angle of the liquid with the solid network [15]. Thus, previous works suggest a solvent exchange from aqueous solution to organic solvents (e.g., hexane) [16,17]. These non-polar solvents are also needed due to the high chemical reactivity of surface modification agents with water, leading to stresses of the materials and insufficient modification of the network [18]. Different surface modification agents lead to differences in the wettability of the gel with water, surface free energies, and thus, altered contact angles of the solvent with the solid backbone [17,19,20]. While aging and strengthening of the network, as well as different gel geometries, can further reduce the overall stresses [4,15,21], these approaches might unintentionally alter the microstructure or macrostructure. It is essential to understand the influence of various surface modification agents on evaporative drying and, therefore, on the final properties of the aerogels.

During APD of aerogels, the gel might shrink up to half its length, followed by an almost complete re-expansion of the material. This phenomenon is called the springback effect (SBE) [11]. This surprising volume change is of great importance in achieving low densities and high porosities [13], and it is directly correlated to the surface modification and hydrophobization of the gel network [11]. On the contrary, insufficient or no surface modification might lead to irreversible shrinkage due to condensation reactions [15]. 

Different silylating agents have been reported in the literature to investigate the APD [19,20,22,23,24,25,26,27,28], with trimethylchlorosilane (TMCS) being the most prominent candidate, and hexamethyldisilazane (HMDS) being an economical substitute. It was shown that TMCS might lead to lower densities, higher hydrophobicities, and lower transparencies than HMDS despite having the same end groups [27]. Furthermore, using TEOS as a precursor, only TMCS lead to monolithic samples; however, with sodium silicate, HMDS became feasible as well [28]. Besides these common additives, other modifying agents, such as dimethylchlorosilane, methyltrimethoxysilane, methyltriethoxysilane, vinyltrimethoxysilane, phenyl triethoxysilane, dimethyldimethoxysilane, and hexamethyldisiloxane, have been researched previously [19,22,29,30,31]. These silylation agents were mainly reported for a silica system, but other inorganic materials, such as alumina, may also be produced by APD [26]. While various surface modification agents have been reported in the past, the research focus was on investigating the APD [22,24,28,32], rather than the SBE, although this phenomenon greatly influences the resulting materials. This is likely due to a lack of monolithic samples being produced, as investigations of the SBE on powders are somewhat limited.

Although it is usually stated that monolithic aerogels can only be achieved by SCD or by creating a fiber composite structure [24,32], diligent solvent exchange and surface modification allow the production of monolithic samples by APD as well [33]. Various surface modification agents were reported to influence the shrinkage of fiber-reinforced gels [22], as the fiber reinforcement prevents [24] or hinders the springback effect [32]. However, it was shown that the SBE of APD specimens might be correlated to the strain recovery observed in their SCD counterparts [34]; drying at supercritical conditions completely negates shrinkage, which conceals the drying behavior of different silylation agents. Therefore, fiber composites, as well as SCD, should be avoided for studying the SBE.

Crack-free monolithic APD silica aerogels modified by TMCS have been produced in the past [11,18,35]. These studies focused on an optimization of the synthesis, such as the molar ratio of TMCS to pore water [11] or TMCS to silica and the effect of the surface modifier on the hydrophobicity [18]. In another work, the effect of the pH of the starting sol was evaluated with respect to the bulk density, porosity, and specific surface area [35]. Other precursors, such as methyltrimethoxysilane, were successfully used to produce monolithic APD silica aerogels, once again optimizing the synthesis parameters [36]. These studies aimed to improve the APD synthesis, focusing on one silylation agent and using the SBE as a performance indicator, rather than as a main subject of interest. 

In this work, an extensive study of different surface-modified dried monolithic silica samples was conducted to investigate the influences of the silylating agents on the springback behavior. Gels with three different surface modification agents, as visualized in Figure S1, i.e., TMCS, HMDS, and triethylchlorosilane (TECS), have been synthesized and compared with the unmodified reference. We expected an additional effect of TECS compared to TMCS, as the bigger molecular size might influence the SBE or the efficiency of modification. The multi-method characterization approach allowed us to show the impact of silylation on the SBE, on a macroscopic and microscopic scale, as well as the chemical environment. We wanted to determine whether indications of the SBE may be found after the completed drying. While we have previously shown that X-ray scattering can be a powerful tool to follow the SBE in situ [37], here it is applied to determine the size of primary particles, which are the elementary structure units. This shows the influences of silylation on monolithic samples from the atomic, to the microscopic, to the macroscopic scale.

## 2. Results and Discussion

Four different sample types, i.e., unmodified (UN) and modified by hexamethyldisililazane (HM), triethylchlorosilane (TE), and trimethylchlorosilane (TM), were investigated by a multi-method approach to show differences in springback behavior due to the various silylation agents. For this purpose, the silica samples were dried by APD for up to five days. Fourier-Transform Infrared Spectroscopy (ATR-FTIR), elemental analysis, nuclear magnetic resonance spectroscopy (NMR), and thermogravimetry analysis (TGA) coupled with mass spectrometry (MS) allowed us to determine the chemical environment of the dried gels. Small-angle X-ray scattering (SAXS) was used to determine the size of primary particles. Additionally, the macroscopic structure was evaluated optically, as well as the microstructure by means of scanning electron microscopy (SEM). Helium pycnometry, X-ray micro-computed tomography (µCT), and nitrogen sorption measurements allowed for a full evaluation of the density, pore structure, and specific surface area of the samples. 

**Figure 1:**
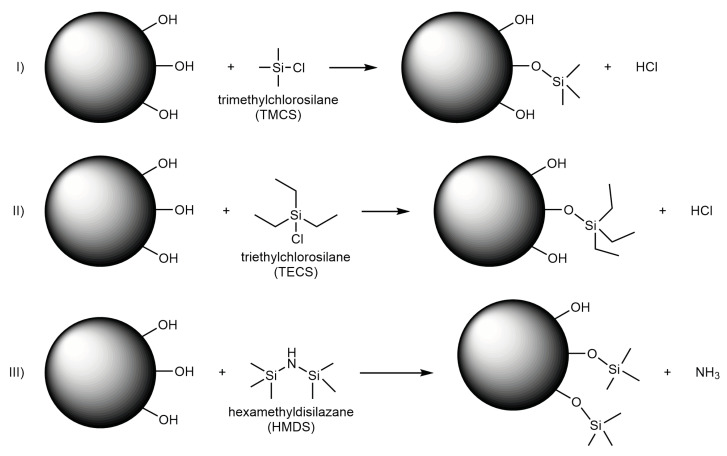
Proposed mechanisms of the silica gel network for (**I**) hydrophobization by trimethylchlorosilane, (**II**) triethylchlorosilane, and (**III**) hexamethyldisilazane. The silanol end groups react with the respective silylation agents, while splitting off either hydrochloric acid or ammonia.

All silylation agents lead to surface hydrophobization by modification with surface silanol end groups, as depicted schematically in Figure 1, splitting off either hydrochloric acid or ammonia. Whereas modification by TECS results in triethylsilyl (TES) end groups, TMCS and HMDS silylation lead to trimethylsilyl (TMS) groups. However, TMCS and HMDS show differences in reactivity [27], and it was reported that HMDS could undergo two different reaction mechanisms during silylation [38]. These differences in reaction mechanisms or steric hindrances because of differences in molecular sizes could lead to influences on the springback behavior.

**Figure 2:**
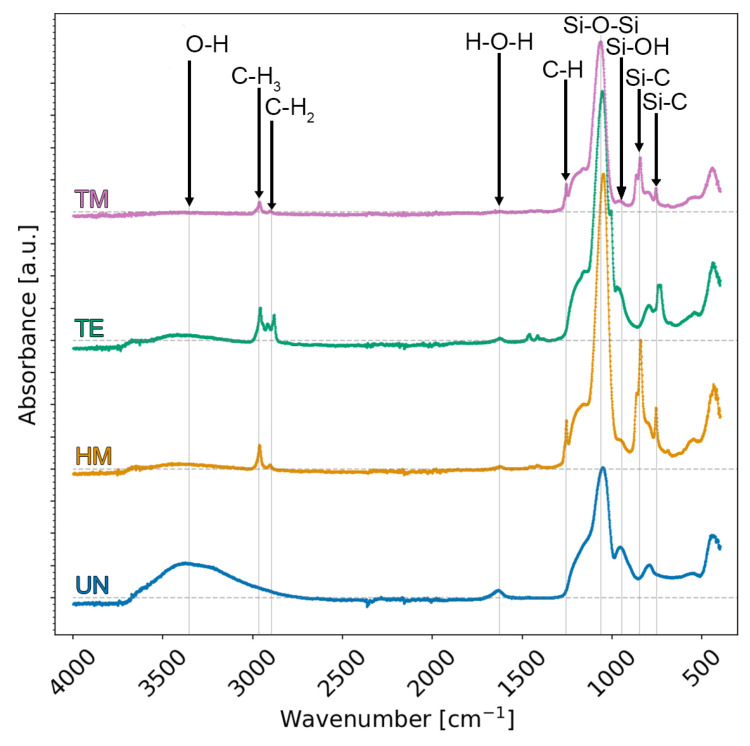
ATR-FTIR spectra were collected on the unmodified UN (blue), hexamethyldisilazane-modified HM (orange), triethylchlorosilane-modified TE (green), and trimethylchlorosilane-modified TM samples (pink) after APD. The modified samples showed typical bands for silylation.

**ATR-FTIR** spectra were collected to prove if the surface modification via TMCS, TECS, or HMDS was successful (Figure 2). All samples showed a Si-O-Si asymmetric stretching vibration at 1100 cm^−1^, as to be expected from a silica structure [22]. A broad absorption band was noticeable at ca. 3500 cm^−1^, as well as an in-plane stretching vibration at ca. 965 cm^−1^, especially for the UN sample, which was negligible for the TM sample but was also partially present for the TE and HM samples. This can be attributed to O-H or Si-OH groups and is, therefore, an indication of unsuccessful surface modification. Similarly, a bending vibration at 1630 cm^−1^ was reported to correspond to adsorbed water [22,33,39]. These results suggest that the TM sample was completely modified. Conversely, the adsorbed water, as well as these Si-OH bands, suggest that the HM and the TE samples were not fully modified, since a silylation would make the surface of the sample extremely hydrophobic [29]. A stretching vibration at 2900 cm^−1^ and a symmetric deformation vibration at 1310 cm^−1^ were related to C-H, and a stretching vibration at 840 cm^−1^ and a peak at 780 cm^−1^, related to Si-C bands, were observed for the TM and the HM samples [22,39]. An additional vibration was observed for the TE sample close to the stretching vibration of the 2900 cm^−1^ C-H band, but it was slightly shifted to lower wavenumbers. This was attributed to methylene bridges [22,33]. In summary, the vanishing of absorption bands corresponding to O-H groups and the observation of characteristic bands of alkyl groups confirm the successful surface modification of the silica aerogel, i.e., the TM sample. However, the absorption bands attributed to the O-H group are not fully vanished for the HM and the TE samples, suggesting the incomplete surface modification of these two samples. 

The carbon and hydrogen content measurements were used to quantify the organic part of the surface modification. Accordingly, the **elemental analysis** with the measured weight percentage wt% of hydrogen and carbon, as well as an equivalent amount of substance mol* in an arbitrary amount of 100 g is summarized in Table 1. Here, the carbon and hydrogen content was noticeably higher for the silylated gels in comparison to the unmodified sample. This is to be expected, as the surface modification introduces carbon- and hydrogen-containing groups (i.e., -CH_3_, -CH_2_CH_3_) to the material surface (Figure 1). As expected, the TE sample showed the highest carbon and hydrogen content, as this sample had ethyl instead of methyl end groups attached. Contrary to the FTIR measurements, the results suggest that there was only a slight difference in surface modification for the TM and HM samples, assuming that the carbon content only arose from the silylation agents. According to the literature, the reactivity of HM should be lower because of the change in pH during the synthesis, as well as a possible two-step reaction [27]. This might be in the range of the resolution of the elemental analysis. Furthermore, all samples showed an undetectable nitrogen content, confirming that the HMDS reacted, as shown in Figure 1, leaving no nitrogen traces inside the HM specimen. Unexpectedly, the carbon content was roughly 1 wt% for the unmodified sample, as only silanol end groups should be present in this material, which could be indicative of residual organic chains of the used solvent. Alternatively, this suggests that the ethoxy end groups of the TEOS precursor remained during the sol-to-gel transformation, and the hydrolysis was incomplete. Considering that this is the only source of carbon content, this would mean for the UN sample that ca. 0.23 mol* of the hydrogen content originated from the ethoxy group (-OC_2_H_5_). The rest of 1.19 mol* would come from adsorbed water (H_2_O) or silanol (-OH) end groups. For simplicity, it may be assumed that the amount of unhydrolyzed TEOS would be identical throughout the different samples, in which case the calculated carbon and hydrogen values of the UN sample could be subtracted from the other specimens. This would result in a carbon content of 1.09 mol*, 1.32 mol*, and 1.09 mol*, as well as a hydrogen amount of 3.42 mol*, 3.47 mol*, and 3.21 mol* for the HM, the TE, and the TM samples, respectively. Considering a successful surface modification with only methyl end groups (-CH_3_) for the HM and the TM samples, this would translate to a H/C ratio of 3, whereas ethyl end groups (-C_2_H_5_) of the TE sample would reach 2.5. Assuming that the remaining carbon solely originated from the silylation of the samples, the remaining hydrogen amounted to 0.14 mol*, 0.18 mol*, and -0.05 mol* for the HM, the TE, and the TM samples, respectively. The hydrogen amount determined for the TM sample was negative, which could be inside the measurement uncertainty, or could indicate that the assumption of an equal amount of ethoxy groups was not valid. Once more, the calculations would suggest a complete surface modification for the TM sample.

**Figure 3:**
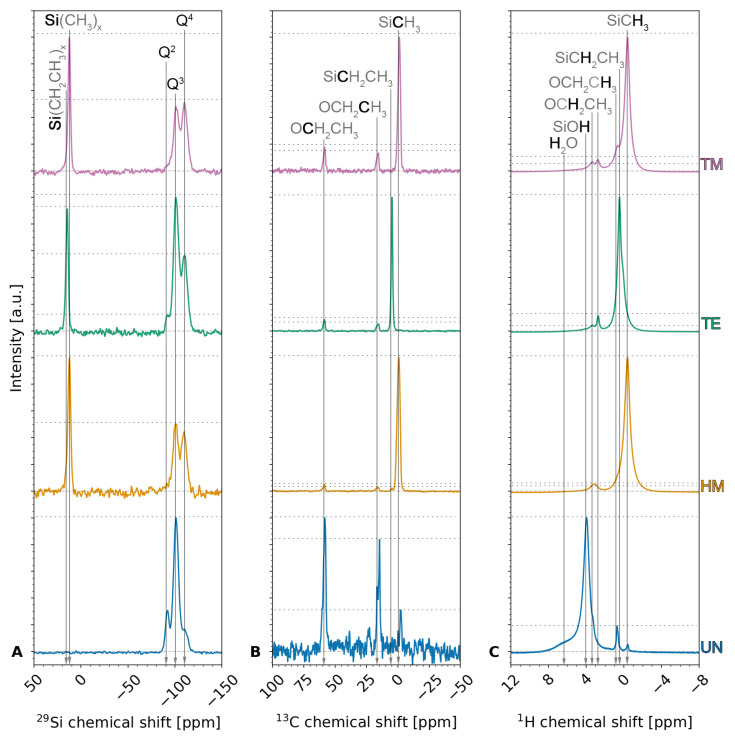
(**A**) ^29^Si, (**B**) ^13^C, and (**C**) ^1^H NMR spectra of the unmodified UN (blue), hexamethyldisilazane-modified HM (orange), triethylchlorosilane-modified TE (green), and trimethylchlorosilane-modified TM samples (pink). A vertical shift was used for better visibility and a grey dotted baseline is shown.

The interconnectivity of the silica network, its chemical environment, and, once more, the species of the silylation end groups can be determined by **^29^Si**, **^13^C**, and **^1^H NMR**. Figure 3 shows the measured ^29^Si (Figure 3A), ^13^C (Figure 3B), and ^1^H NMR (Figure 3C) spectra measured for the four samples, whereas Figures S2 and S3 visualize the calculated ^13^C and ^1^H spectra of TEOS, and HMDS, TECS and TMCS, respectively. Furthermore, the Gaussian peak areas of the ^29^Si, ^13^C, and ^1^H NMR were calculated and are summarized in Tables S1–S3, respectively.

**^29^Si NMR** was used to assess the interconnection of the silica network. The measurements showed one peak at a chemical shift of ~12 ppm for the samples modified with a methyl end group, i.e., the HM and TM samples, and at ~14 ppm for the TE sample modified with an ethyl end group. This peak, which is not observed for the unmodified UN sample, can be attributed to the TMS and TES groups [33,40,41]. Peaks at −92 ppm, −100 ppm, and −110 ppm were visible for all samples and were attributed to Q2 [41,42], Q3 [40,41], and Q4 [33,40,41], respectively, where Qn denotes the amount *n* of bridging oxygen (BO) and *n* − 1 of non-bridging oxygen (NBO) for a tetrahedral silicon conjunction [40]. Here, NBO could be associated to either hydroxyl or methyl groups [42,43]. Comparing the Gaussian peak areas for the UN sample, a qualitative ratio of 16.6%, 69.8%, and 13.7% was seen for Q2, Q3, and Q4, respectively, showing a high contribution of silicon conjunctions with three BO and one NBO. These results indicate the high amount of hydroxyl groups on the surface of unmodified UN silica xerogels. On the contrary, the silylated samples show a ratio of 7.2%, 26.9%, 29.2% for the HM sample, 3.7%, 45.7%, 29.9% for the TE sample, and 4.1%, 27.7%, 37.0% for the TM sample. The rest of these ratios comprised the TMS/TES peak with 36.6%, 20.7%, and 31.3%, respectively. Here, the position of the TMS peaks confirms that the HM and the TM samples are of the same species of silyl end groups. The decrease in the Q3 species and increase in Q4 species after modifying the surface modification can be explained by the chemical reaction between Si-OH (Q3) and Cl-Si(CH_3_)_3_ or Cl-Si(CH_2_CH_3_)_3_ to form Si-O-Si (Q4). These results suggest the successful modifications of the silica aerogels/xerogels, and the highest degree of modification is observed for the TM sample, as indicated by the highest Q4 percentage. A high contribution of Q3 for the TE sample was noticeable, which might be explained by the steric hindrance of the bigger TECS molecule. These results agree with FTIR results, as discussed above.

**^13^C NMR** was used to gain insight into the chemical environment of the carbon atoms, which should mainly arise from surface modification. The ^13^C NMR measurements displayed peaks for all samples at chemical shifts of ~58 ppm and ~16 ppm, which can be attributed to -CH_2_- (57.8 ppm–59 ppm) and -CH_3_ (15 ppm–17.9 ppm) units of ethoxy end group -O-CH_2_-CH_3_, indicating incomplete hydrolysis of TEOS during the synthesis [33,39,44,45] and thus confirming the results of the elemental analysis. This is in agreement with the values calculated for pure TEOS (Figure S2). The qualitative results of the ^13^C measurements suggest a high contribution of incomplete hydrolysis for the UN sample compared to the silylated HM, TE, and TM samples. Surprisingly, this contribution was noticeably higher for the TE and TM samples than for the HM sample. Contrary to the assumptions for the elemental analysis, these results might suggest that the amount of unhydrolyzed ethoxy groups was not identical for the different silylation agents. Thus, this would mean that the silylation agents, i.e., HMDS, TECS, and TMCS, reacted with the unhydrolyzed ethoxy groups. The UN, HM, and TM samples showed a peak at ca. −1 ppm that was once more attributed to TMS groups [33,41,45]. Likewise, the TE sample showed a peak at 5 ppm, slightly shifted from the methylene peak reported in the literature at 10.9 ppm [46], confirming the TES groups. Unexpectedly, the UN sample also showed a contribution of TMS groups, which was described in a previous study and associated with gas phase deposition since the unmodified sample was dried in proximity to the silylated samples [41]. In this work, this explanation is highly unlikely because the samples were rinsed multiple times after silylation and before drying. Furthermore, the samples were not in close proximity during drying. These TMS groups might be explained by impurities in TEOS. Finally, the ^13^C NMR measurements confirmed the incomplete TEOS hydrolysis and showed relevant peaks of the silyl end groups.

The **^1^H NMR** spectra were measured to determine the chemical environment of the hydrogen atoms, thus indicating the completeness of the surface modification. The spectra showed peaks at ca. 6 ppm and roughly 4 ppm for the UN sample, which were likely attributed to adsorbed water [42] and silanol end groups [47,48]. It was reported that the silanol peak would usually be very weak compared to the methyl group [49]. Here, the qualitative ^1^H NMR analysis confirms a high contribution of silanol end groups. One or two peaks of the methylene group as well as a peak from the methyl of the ethoxy end group were noticeable around 3 ppm and 1 ppm, respectively [41,42,50]. Whereas the UN, the HM, and the TM sample showed a peak close to 0 ppm, which was associated with TMS [41,42,50], this peak was slightly shifted to higher values for the TE sample, likely displaying the methylene group. Once more, this confirmed that the UN sample inhibited TMS groups. Furthermore, the qualitative ^1^H NMR confirmed the qualitative ^13^C measurements by demonstrating a higher contribution of ethoxy groups for the TE and TM samples than the HM sample. Two small peaks at roughly −25 ppm and 25 ppm were seen but not shown here, and they are attributed to spinning sidebands. In conclusion, signs of incomplete TEOS hydrolysis were found once more in all samples; the UN sample showed a high contribution of silanol end groups and absorbed water, while silylation was confirmed for the HM, TE, and TM samples.

**Figure 4:**
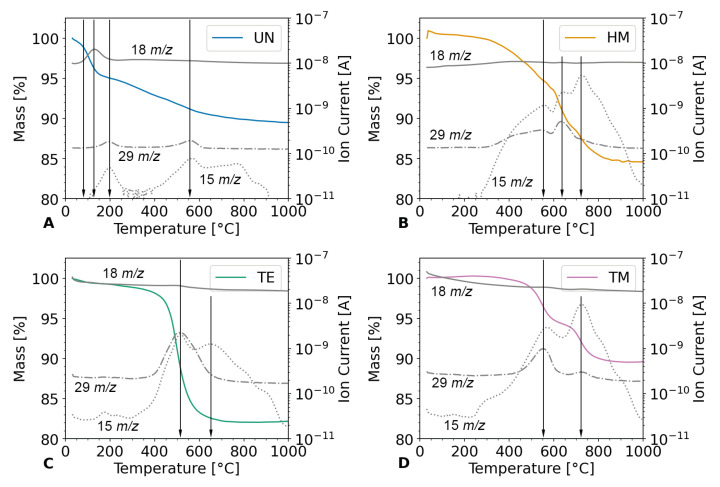
TGA measurements coupled with MS for the (**A**) unmodified UN (blue), (**B**) hexamethyldisilazane-modified HM (orange), (**C**) triethylchlorosilane-modified TE (green), and (**D**) trimethylchlorosilane-modified TM samples (pink). The ion current of the MS measurement is shown for selected molecular weights of 18 *m*/*z* (solid), 15 *m*/*z* (dot), and 29 *m*/*z* (dash-dot). A vertical black line was drawn to highlight changes in the MS measurements.

Consequently, the thermal stability of the specimen as well as their chemical species was evaluated. To this end, **TGA** was **coupled** with **MS** (Figure 4), relating the weight loss to the released gas species. The weight loss of the samples was ca. 11%, 16%, 18%, and 11% for the UN, HM, TE, and TM samples, respectively. This higher weight loss for the HM and TE samples was expected in comparison to the UN sample, since the organic end groups of the surface modification already showed a high contribution in the elemental analysis. The weight loss for the HM and TE samples correlated with the calculated weight of silyl end groups of the elemental analysis. The relatively low weight loss for the TM sample was surprising but could be explained with a different sample geometry. Here, the measurement was repeated because of implausible data, which was likely caused by the light powder being sucked out of the crucible by the carrier gas. Thus, for this sample, the measurement was performed on a monolithic sample of the same synthesis batch, whereas the other samples were in powder form and investigated with multiple methods on the same specimen. The UN sample showed a weight loss of about 2% at roughly 130 °C that can be attributed to the evaporation of adsorbed water residue on the surface, as indicated by the detection of H_2_O (*m*/*z* = 18) in the MS spectra, succeeded by further weight loss of ca. 3%. This was attributed to the evaporation of water, followed by condensation reactions of the hydroxyl end groups, as indicated by the literature [51]. Additional weight loss of about 6% was observed in the temperature range of 150–570 °C and accompanied by the evolution of methyl (CH_3_ *m*/*z* = 15) and ethyl (C_2_H_5_ *m*/*z* = 29) gas species, indicating the thermal decomposition of unhydrolyzed ethoxy groups of the TEOS precursor. In contrast to the unmodified UN sample, neither significant weight loss nor water was detected in the TG and MS of the HM, TE, and TM samples below 200 °C, suggesting the negligible amount of adsorbed water in these samples. All modified samples showed a significant weight loss above 400 °C, accompanied by the evolution of methyl (CH_3_ *m*/*z* = 15) and ethyl (C_2_H_5_ *m*/*z* = 29) gas species, suggesting the thermal decomposition of methyl and ethyl groups that resulted from the surface modifications. According to the literature, the decomposition of surface methyl groups may happen between 300 °C and 500 °C [52,53,54] and is strongly dependent on the gas atmosphere [31]. As expected, a higher amount of ethyl gas species was detected for the TE specimen modified with TECS, while the HM and TM samples modified with TMCS and HMDS, respectively, released a higher amount of methyl gas species. These results indicate that the thermal decomposition of the ethyl groups in the TE sample is completed at 600 °C, which is much lower than the temperature (~800 °C) required to complete the decomposition of methyl groups in the HM and TM specimen. Although the TM sample showed the same weight loss as the UN sample, there was no adsorbed water detected in the TM sample, and all weight loss is attributed to the thermal decomposition of the methyl group that resulted from the surface modification. This stepwise oxidation of methyl groups was reported in the literature [55]. In summary, the thermal analysis results confirm the surface modification of the silica aerogels, which is stable up to 300 °C.

Nanoscopic structural changes of the specimen, e.g., the size of primary particles, the fractal dimension, and other parameters, can be elucidated by means of **SAXS** measurements. Scattering data provide information about the size of primary particles, which are the elementary structure units and backbone of the material, by analyzing the SAXS profile in a region between the fractal dimension and Porod slope [56]. This fractal region represents a branched network with either structural or mass self-similarity [57]. By correlating the fractal dimension with computer models, it is possible to gain insight into the network formation during the sol-gel transition [58,59]. Similarly, the Porod slope provides insight into the interfacial roughness of a material [60]. To this end, SAXS data were evaluated, assuming a fractal model with spherical elementary particles, as described by Teixeira et al. [61], able to provide information about the size of primary particles and the organization of the aerogel skeleton assuming a fractal organization. The measurements, as well as the model fits, are shown in Figure S4, while the calculated parameters are visualized in Table S5. Fitting models were obtained by assuming a static radius polydispersity. The fractal dimension was determined to be 3.42, 2.64, 2.71, and 2.57 for the UN, HM, TE, and TM samples, respectively. Accordingly, radii of primary particles were estimated, showing values of ca. 3.2 Å for the UN sample, 4.0 Å for the HM sample, 3.5 Å for the TE sample, and 4.9 Å for the TM sample. These results suggest slightly larger primary particles for the TM sample compared to the HM, TE, and UN samples, declining in this order. Previous studies reported a diameter of primary particles of approximately 10 Å, which might vary according to the synthesis conditions [56]. Before surface modification, all samples should show the same size of primary particles, which form during the initial hydrolysis and condensation reactions. These calculations reflect the dried gel samples. Overall, the SAXS measurements suggested an influence of the silylation on the fractal dimension, i.e., the network formation, as well as the size of elementary spherical particles. 

**Figure 5:**
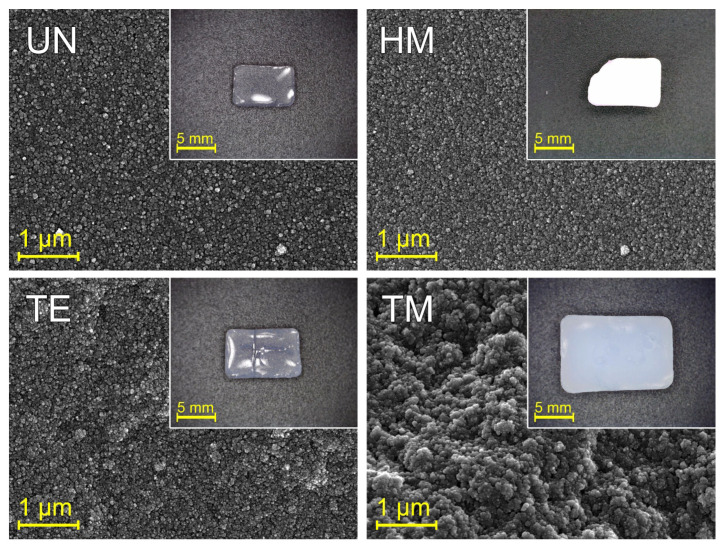
SEM images of the unmodified UN (top left), hexamethyldisilazane-modified HM (top right), triethylchlorosilane-modified TE (bottom left), and trimethylchlorosilane-modified TM samples (bottom right) after APD drying showed differences in their microscopic structure. While all displayed spherical particle morphologies, the size was bigger for the TM in comparison to the UN, the HM, and the TE samples. Additionally, inlets are shown with photographs of the respective monoliths, showing a decrease in size in the order of the TM, the TE, the HM, and the UN samples.

To put the size of the smallest elements of the structure into context with the microstructure of the samples, **SEM** (Figure 5) was used. While the UN, HM, and TE samples demonstrated a relatively dense structure consisting of small particles, the TM sample showed larger pores with interconnected bigger particles in comparison. However, all samples seemed to consist of particles with nearly spherical shapes. Little differences between the UN, HM, and TE samples could be found, suggesting that silylation agents do not necessarily lead to an altered microstructure. 

Following this, the influence of silylation on the re-expansion was investigated. The insets of Figure 5 show the **digital images** of these four sample types after APD drying, and they were used to document the SBE. Size differences were noticeable, where the TM sample was the largest, followed by the TE, the HM, and finally, the UN sample. Furthermore, the specimen showed remarkable differences in transparency and color. The HM sample was white opaque, whereas the UN and TE samples displayed transparency. The TM sample exhibited translucency with the typical blueish color due to Rayleigh scattering [40]. However, these macroscopic changes differ within various synthesis batches (see Figure S5). These changes could be due to differences in lighting conditions, affected by micro-cracks inside the material, or slight changes in room temperature and, thus, drying velocity. While some samples did not endure the shrinkage and re-expansion and, for this reason, broke into pieces, the size of the samples seemed to show the same trend within one sample type. This leads to the belief that the differences in synthesis batches show the same trend of springback behavior. Similar to the results of the SEM, silylation did not strictly lead to a fully re-expanded structure; therefore, differences in the SBE were observed.

**Figure 6:**
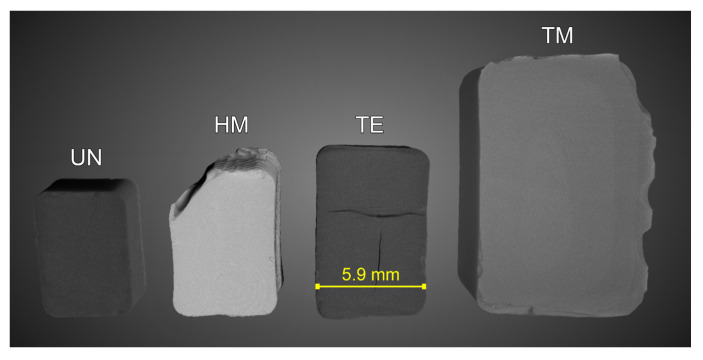
The samples as they appear in the Dragonfly software after segmentation. From left to right, the unmodified UN, hexamethyldisilazane-modified HM, triethylchlorosilane-modified TE, and trimethylchlorosilane-modified TM samples are shown. One dataset from each surface modification batch was selected. Additionally, a scale bar was added for the TE sample for visualization.

**Figure 7:**
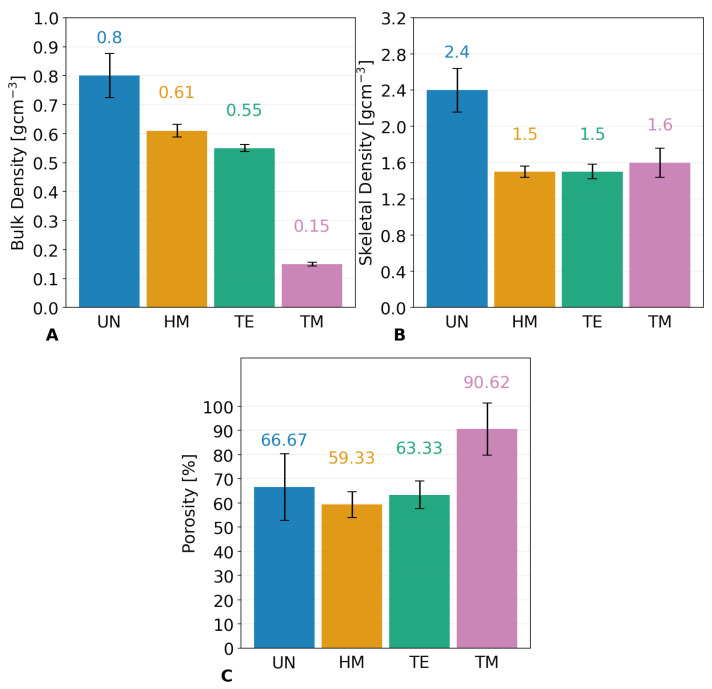
Evaluations of the unmodified UN (blue), hexamethyldisilazane-modified HM (orange), triethylchlorosilane-modified TE (green), and trimethylchlorosilane-modified TM (pink) samples for (**A**) the bulk density measured by µCT; (**B**) the skeletal density determined by helium pycnometry; (**C**) the porosity calculated from the measurements of the bulk and skeletal density shown with their respective errors. The measurements show decreasing bulk densities in the order of the UN, the HM, the TE, and the TM samples, indicating a strong influence of surface modification.

Additionally, the SBE and re-expansion of the monoliths can be confirmed by their bulk density. The bulk density was determined by means of **µCT** measurements to calculate the volume of the UN, HM, TE, and TM samples on three dried samples each, as well as measuring their weight. Segmentation of the µCT data was carried out excluding cracks larger than ca. 60 µm, thus providing a distinct advantage over powder pycnometry commonly used to determine the bulk/envelope density [14,34,40], while being more precise than estimating the volume from optical images. Figure 6 shows one segmented 3D volume for each sample type. Furthermore, Figure S6 displays one 2D slice with a scale bar and additional information for the UN, HM, TE, and TM samples, respectively. The µCT measurements confirmed the differences in width previously mentioned for the optical images, showing values of ca. 9 mm, 5.9 mm, 5.4 mm, and 4.6 mm for the TM, TE, HM, and UN samples. Accordingly, the gel volume was, respectively, 542.4 mm^3^, 173.0 mm^3^, 140.5 mm^3^, and 86.1 mm^3^, as displayed in Figure 6. However, it was assumed that the top left of the HM sample is broken, thus making a measurement of the width more reliable to compare the overall springback behavior. Since an excess in silylation agents during synthesis was used for all samples and the volume before drying was roughly equal, this leads to a belief that the re-expansion due to the springback effect is severely higher for TMCS, followed by TECS and lastly HMDS. Different pore volume/bulk density and, thus, springback behavior of TMCS/HMDS have been reported in the literature [62]. This might be due to differences in reactivity, since it was assumed that the increase in pH because of the ammonia release during the HMDS modification was detrimental, contrary to a decrease in pH during the TMCS modification [27]. Additionally, it was suggested in the literature that HMDS might undergo a two-step reaction [38], which could lead to a decrease in reactivity [27]. Furthermore, the ingress of HMDS in the pores might be hindered by its higher molecular weight, and thus, steric hindrance could lead to insufficient surface modification [27,63]. Since TECS has a higher molecular weight than TMCS, the lower SBE for the TE sample could be attributed to steric hindrance. Additionally, although all surface modifications were done with identical volume ratios, the molarity of the excess mixtures of silylation agent and hexane declined in the order of TMCS, TECS, and HMDS. These results are in good agreement with FTIR, the elemental analysis, and the NMR results, which revealed the highest degree of surface modification for the TM sample. Figure 7A shows the calculated bulk densities for the four samples. The UN, HM, TE, and TM samples showed values of 0.80 gcm^−3^, 0.61 gcm^−3^, 0.55 gcm^−3^, and 0.15 gcm^−3^, respectively. The TM sample differed severely from the other three samples, especially the UN sample. This was expected, as the bulk density is mainly influenced by the re-expansion of the material, assuming that the volume of the samples was similar before drying. In conclusion, the silylation always increased the sample volume and decreased the bulk density but was noticeably different for the HM and TE sample in comparison to the TM sample, which showed properties typical for an aerogel.

**Figure 8:**
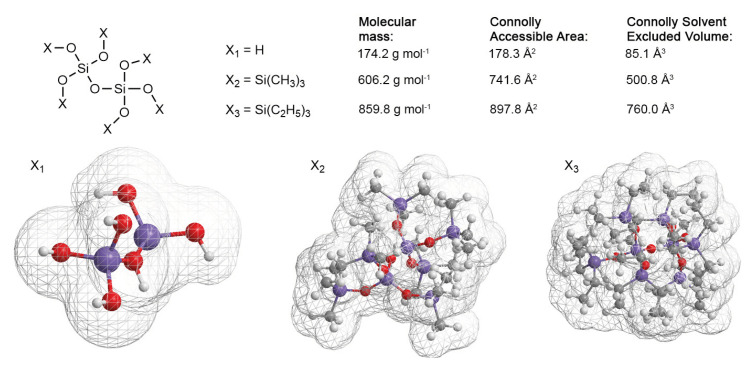
Sketch of the influences of a surface modification on an unmodified sample (X1), via TMCS or HMDS (X2) or TECS (X3) on the overall molecular structure. Here, a chain with two silica atoms is shown with the calculated molecular masses, accessible areas, and Connolly volume inaccessible by a solvent with a radius of 0.5 Å. The grid of the 3D structures represents the volume inaccessible by this solvent. This illustration is a simplification and values should be considered with care.

The skeletal density was measured by **helium pycnometry** to determine the influence of silylation on the backbone of the structure, ultimately allowing us to calculate the porosity of the sample. Contrary to the bulk density measurements (Figure 7A), here, the three samples were crushed to a powder to improve the measurement uncertainty. The results are shown in Figure 7B. Whereas the UN sample was measured to be ca. 2.2 gcm^−3^, all three modified samples were determined to be roughly 1.5/1.6 gcm^−3^. However, the expanded measurement uncertainty was 0.24 gcm^−3^, 0.06 gcm^−3^, 0.08 gcm^−3^, and 0.16 gcm^−3^ for the UN, HM, TE, and TM samples, respectively, which might be improved by a higher sample volume. Noteworthy was the similarity for all surface modifications, as well as the significant difference of 0.6 gcm^−3^ to the unmodified sample. Values of 1.9–2.2 gcm^−3^ are often used in the literature for calculations, e.g., of the porosity [14,54,64,65]. One common artifact in helium pycnometry measurements is the effect of closed pores inside the specimen [22]. Since the wet gel volume of surface-modified and unmodified samples should be roughly the same before drying, and the unmodified sample shows irreversible shrinkage in comparison to the other samples, closed porosity is highly unlikely to be the reason for this discrepancy in skeletal densities, as this decrease, because of closed porosity, should either be even more pronounced for UN or at least similar. Furthermore, it was reported that there is a direct correlation between structure modification and a decrease in skeletal density [31,66]. Figure 8 illustrates this phenomenon for a small silica gel. This simplified structure was shown here with silanol and two different silyl end groups. Their volume was determined in Chem3D using a Connolly solvent excluded volume calculation [67]. While the silanol end groups of the unmodified material are relatively small, the silylation introduces longer and lighter organic chains in comparison to the silica backbone, increasing the overall volume inaccessible by the helium molecules during pycnometry, while not gaining molecular mass proportionally. This means that the same mass of two structures would occupy less volume for an unmodified sample in comparison to the modified sample, thus increasing its skeletal density. Consequently, the decrease in skeletal density due to the surface modification should be even more pronounced for the TE sample, as it introduced even longer organic chains but could not be verified by the helium pycnometry measurements. This might be caused by insufficient modification of the surface, as confirmed by the prior measurements, or could lay inside the error margin of the measurement. As previously mentioned, this insufficient modification could be triggered by steric hindrance of the silylation, as can be seen in the simplified model of Figure 8, where TECS shows overlapping chains of molecules. In summary, the skeletal density decreased heavily for all surface-modified samples, i.e., the HM, TE, and TM samples, and was seemingly not influenced by the SBE.

The porosity (Figure 7C) was calculated from the bulk and skeletal densities (see Equation (1)). As expected, the porosity was highest for the TM sample, which showed the largest re-expansion of the material and, thus, the greatest springback effect. A porosity of 66.67%, 59.33%, 63.33%, and 90.62% was determined for the UN, HM, TE, and TM samples, respectively. Surprisingly, the re-expansion among other samples was determined to be similar, where the HM and TE samples should show slightly higher porosities than the UN sample because of their slight re-expansion. Though the differences in skeletal densities could be an explanation for this discrepancy, this is most likely to be attributed to error margins within both density measurements and will therefore be disregarded. Within the measurement, only the TM sample could be considered an aerogel.

**Figure 9:**
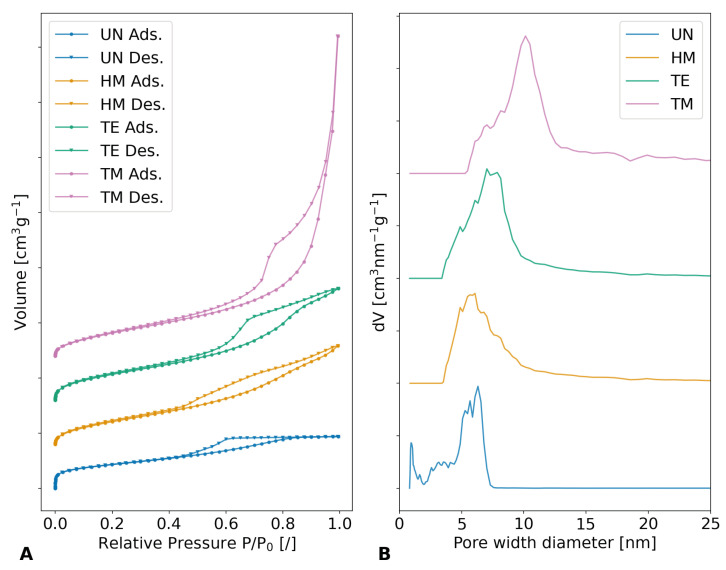
Results with a vertical shift of the unmodified UN (blue), hexamethyldisilazane-modified HM (orange), triethylchlorosilane-modified TE (green), and trimethylchlorosilane-modified TM (pink) samples of (**A**) nitrogen adsorption (Ads.) and desorption (Des.) isotherms, as well as their respective specific surface areas; (**B**) the NLDFT evaluations with equilibrium model and cylindrical pore geometry. The pore width diameter showed an overall decrease in the order of the TM, the TE, the HM, and the UN samples, while a noticeable change in isotherms was visible.

The influence of different silylation agents on porosity can be supported by gas adsorption measurements, which should show different pore diameters and volumes. The **nitrogen isotherm measurements**, which are shown in Figure 9A as well as Figure S7, show severe differences in their adsorption volume and behavior and were used to calculate the pore diameter and volume. The samples showed a typical type IV(a) isotherm with hysteresis, which was reported for mesoporous materials [68]. The evaluation of the specific surface area according to Brunauer–Emmet–Teller (BET) unveiled values of 767.5 m^2^g^−1^, 852.7 m^2^g^−1^, 930.5 m^2^g^−1^, and 902.5 m^2^g^−1^ for the UN, HM, TE, and TM samples, respectively. The higher specific surface areas for the TE sample in comparison to the TM sample could be explained by a shift of the pore width diameter distribution to lower values, since lower pore widths contribute greatly to the specific surface area but not the pore volume. Furthermore, their total adsorbed volume differed, declining in the order of the TM, TE, HM, and UN samples. All samples demonstrated a sharp rise at low relative pressures, suggesting the presence of microporosity. This increase was more prominent for the HM and the TE than the TM sample and even more pronounced for the UN sample. This is to be expected, as during the drying of the samples, the pore structure collapses and the overall pore size distribution shifts to lower values. During the springback effect, a part of this collapse is reversed and the pore size is recovered. Figure 9B and Figure S8 show the results of the non-local density function theory (NLDFT) for determining the pore size distribution. Once more, a trend in the order of TM, TE, HM, and UN samples was calculated, showing a decrease in average pore width diameter, as well as the cumulative pore volume. The maxima of the pore width diameter/cumulative pore volume were determined to be roughly 10.1 nm/3.13 cm^3^g^−1^, 7.0 nm/1.5 cm^3^g^−1^, 6.1 nm/1.3 cm^3^g^−1^, and 6.3 nm/0.7 cm^3^g^−1^ for the TM, TE, HM, and UN samples, respectively. While the UN sample showed a slightly higher maximum in pore width diameter than the HM sample, the size distribution was more inhomogeneous for the unmodified specimen, showing a significant contribution of micropores. Overall, the trend of the nitrogen sorption measurements was the same as for the bulk density measurements and porosity evaluations, showing higher pore volumes and pore diameters for modified samples with more pronounced SBE.

The specific pore volumes and mean pore diameters can be calculated using the bulk density, skeletal density, and specific surface area, as described in Equations (2) and (3). These findings, as well as the nitrogen sorption and NLDFT evaluations, have been summarized in Table 2. In line with the NLDFT calculations, the specific pore volume and mean pore width values decrease in the order of the TM, TE, HM, and UN samples. Here, the TM sample showed noticeably higher values, which might be attributed to a higher content of meso- and macropores inside the material, which have a high impact on pore volume and mean pore width but negligible effect on the specific surface area of a material. 

## 3. Conclusions

**Figure 10:**
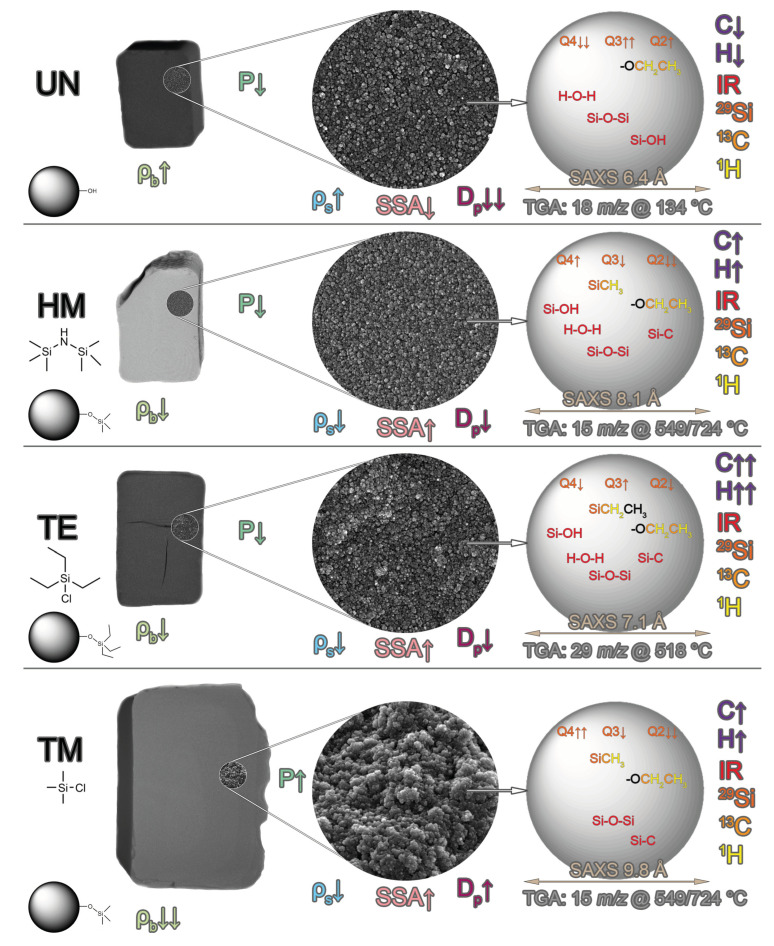
The main differences in drying behavior, chemical composition, density, porosity and microstructure between the unmodified UN, hexamethyldisilazane-modified HM, triethylchlorosilane-modified TE, and trimethylchlorosilane-modified TM silica gels. The chemical formulas of HMDS, TECS, and TMCS are visualized underneath HM, TE, and TM, respectively. Each sample shows the macroscopic structure as captured by µCT and the microscopic structure by SEM, as well as measured information about the primary particles. The comparison of the measured parameters is displayed as an arrow for each sample. From left to right, the following information is shown: bulk density ρ_b_ (light green), porosity P (green), skeletal density ρ_s_ (blue), specific surface area SSA (pink), pore size D_p_ (magenta), carbon and hydrogen elemental analysis (purple), detected FTIR groups (red), detected ^29^Si (dark orange), ^13^C (orange), and ^1^H (yellow) NMR spectra, primary particle size by SAXS (brown), and main TGA weight loss (grey).

In this work, we have demonstrated that surface silylation significantly influences the drying behavior of silica gels. For that purpose, the unmodified UN, hexamethyldisilazane-modified HM, triethylchlorosilane-modified TE, and trimethylchlorosilane-modified TM monolithic samples were synthesized and characterized by several complementary methods. Figure 10 summarizes the main difference between the studied specimens with respect to their drying behavior, chemical composition, density, porosity, and microstructure. Additionally, the majority of parameters are shown in Table S6.

Without any surface modification, the UN sample condensed irreversibly, shrinking to small cuboid geometries, leading to an increased bulk density and relatively low porosity. The specimen consists of small spherical particles in a comparatively dense structure, with small pores and a low pore volume. The primary silica particles, roughly 6 Å in diameter, constitute the silica backbone with a majority of silicon atoms with three bridging oxygen (BO) and one non-bridging oxygen (NBO). Some ethoxy groups were present because of incomplete hydrolysis, but the sample was mostly composed of silanol end groups. This resulted in a significant amount of ca. 5 wt% adsorbed water. 

The HM and TE samples were used as a comparison to the unmodified UN and the TMCS-modified TM samples. While the modification with HMDS and TECS for the HM and TE samples was confirmed, the macroscopic structure did not change significantly. Although the macroscopic size of the HM and TE samples was a bit bigger than the UN sample, both showed irreversible densification and minor re-expansion, which was insignificant on the microscopic scale. This had a minor effect on the bulk densities and slightly increased the average pore diameters and pore volumes. The overall porosity did not change significantly compared to the UN sample, as the surface modification decreased the skeletal density of the HM and TE samples. The diameter of the primary silica particles was roughly 7 Å and 8 Å for the TE and HM, respectively, and the silica backbone was changed drastically. The amount of Q3 silicon atoms decreased with respect to the Q4 content, suggesting a higher content of BOs. This change was even more pronounced for the HM sample. Nonetheless, FTIR, NMR, and the elemental analysis indicated the remaining silanol end groups for both samples. The thermal stability of the silylation was determined to be lower for the TE sample compared to the HM sample.

The TM sample was the only material that may be called an aerogel. It showed significant re-expansion, which was confirmed by its large decrease in bulk density. Not only did the macroscopic size change, but also the microscopic morphology displayed larger interconnected particles with bigger pore sizes. Thus, the pore volume increased drastically. Even though the skeletal density was comparable to the other surface-modified samples, the TM sample had the highest porosity. Additionally, the primary particle diameter was calculated to be slightly higher at ca. 10 Å. The silica backbone had the highest content of Q4 silicon atoms and, thus, the highest degree in BOs. Once again, an indication of incomplete hydrolysis was found with a low amount of methylene and ethoxy groups. The thermal stability of the surface modification was similar to the HM sample, completing the decomposition at around 800 °C, compared to the TE sample at roughly 600 °C.

The SBE is governed by the amount of silanol end groups, as condensation reactions lead to irreversible densification [15]. To study this phenomenon, monolithic samples are needed [11], without restricting the shrinkage or re-expansion by a fiber reinforcement [22,32] or by SCD [15]. In the case of no surface modification, the silica gel shrinks, which results in a small sample with high bulk density. The modification by TECS and HMDS did not lead to a pronounced springback effect, and only a slight re-expansion was observed. On the contrary, the TMCS modification was successful in producing monolithic APD silica aerogels, showing a high degree of re-expansion. A comparison of TEOS and sodium silicate precursors could be worthwhile, as the latter was reported to produce a monolithic specimen by silylation with TMCS as well as HMDS [28]. Though the HM and the TE samples presumably had a higher content of silanol end groups than the TM sample, this difference was relatively small. This leads us to believe that even these slight differences are important for the springback effect, or rather that the degree of surface modification is not the only cause. Steric hindrance of the HMDS and TECS could be another influence, where smaller pores of the silica structure might prevent the ingress of these silylation agents due to their molecular size, potentially introducing inhomogeneities of the surface in comparison to the porous network of the sample. Nonetheless, for the HM and TM samples, the silyl end groups should be of the same species. The interconnectivity of the samples could be another important factor, where the amount of BO and NBO is seemingly correlated with the degree of re-expansion. However, it is difficult to conclude that the interconnectivity influenced the SBE and not vice versa. The drying behavior of the samples might also be investigated in situ by means of X-ray scattering, since structural features of the material correlate with the SBE, as we have shown in the past [37]. Further studies are needed to fully understand this behavior.

## 4. Materials and Methods

### 4.1. Materials

Tetraethyl-orthosilicate (TEOS, ≥99%, Alfa Aesar) was ordered from VWR. Hydrochloric acid (37%), ammonium hydroxide solution in water (25%), trimethylchlorosilane (TMCS, purified by redistillation >99%), triethylchlorosilane (TECS, 99%), and hexamethyldisilazane (HMDS, reagent grade ≥99%) were obtained from Sigma-Aldrich (Merck, Germany). Hexane (n-hexane, >99%) and ethanol (>99.5%, Ph.Eur., reinst) were acquired from Carl Roth. Furthermore, ethanol (≥96% denatured, GPR RECTAPUR^®^) was ordered from VWR for solvent exchange. Deionized water (DIW) was used for the synthesis. 

### 4.2. Sample Preparation

The synthesis of silica gels with various surface modifications was performed as adapted from Wei et al. [33]. A total of 6.01 g of silica (9.35 wt% of the final gel) was produced as follows: 20.84 g of TEOS was mixed with 8.78 mL of ethanol, as well as 8.77 mL of an ethanol/37% hydrochloric acid solution (438.19 mL/105 µL). Then, 1.81 mL of DIW was used to form a sol. This solution was stirred for 90 min. Afterwards, 29.23 mL of ethanol and 4.69 mL of a DIW/25% ammonium hydroxide solution (168 g/1 g) were added, and the solution was stirred for 45 min. This solution was left to gel in custom-made Teflon molds, where one gap was 1.5 cm by 1 cm by 0.6 cm. Aging of the cuboid samples was conducted for 24 h at 50 °C.

Afterwards, a solvent exchange was performed with an excess of ethanol, mixtures of ethanol and hexane (25 vol%/75 vol%; 50 vol%/50 vol%; 75 vol%/25 vol%), and four times pure hexane for 24 h, each at room temperature. The surface modification was done under equal conditions for the different silylation agents, using subsequently a 3 vol% and 6 vol% solution of silylation agent in hexane, repeating each step twice, for a total of four individual steps. The gels were modified using TMCS 108.64 gmol^−1^ (denoted as TM), TECS 150.72 gmol^−1^ (denoted as TE), and HMDS 161.39 gmol^−1^ (denoted as HM), respectively. This translates to molarity of 0.236 M, 0.179 M, and 0.144 M of TMCS, TECS, and HMDS hexane mixtures for the 3 vol% solutions, as well as 0.473 M, 0.357 M, and 0.288 M for the 6 vol% solutions, respectively. Residues of the surface modification were rinsed with hexane four individual times at roughly 24 h intervals. Some samples were left unmodified (denoted as UN) and used as references. Finally, the samples were left to dry at moderate evaporation speeds of up to three days.

### 4.3. Methods

Optical images were acquired by means of digital light microscopy (TOOLCRAFT USB microscope 5 MP) to evaluate macroscopical changes. The surface morphology was visualized with scanning electron microscopy (SEM) using a LEO Gemini 1530 (Zeiss, Germany) at 3 kV with a 30 µm aperture and InLens detector. To avoid surface charging, the samples were coated with a thin layer of carbon (Emitech K550 Carbon coater).

The bulk density was calculated from the weight and the volume of selected dry gels. Three gels of each sample type were evaluated for a total of twelve samples. The weight of the gels was measured by an analytical balance PCE-AB 100 (PCE Deutschland GmbH, Meschede, Germany) and the volume by X-ray micro-computed tomography (µCT). The measurements were carried out on an EasyTom 160/150 (RX Solutions, Chavanod, France) equipped with a micro-focus tube (tungsten filament) and a flat panel detector (CsI scintillator). The scans were performed with a voxel size of 9.45 µm in the middle focal spot mode at a voltage of 100 kV and a current of 100 µA. A total of 1440 filtered back projections were acquired in the step and shot mode with reference images. The vertical stack of images was reconstructed with a cone beam algorithm in XAct software (RX Solutions). Dragonfly software (v 4.1; Object Research System Inc., Montreal, Canada) was used to segment the images and extract the volume of the gels.

The skeletal density was determined for the same samples by means of helium pycnometry, using an AccuPyc II 1340 (Micromeritics Instrument Corporation). A 3.5 cm^3^ cup was used, and each measurement was preceded by rinsing the chamber ten times. The measurements were repeated until a constant sample volume was determined. The calibration of the instrument was performed and confirmed with a certified spherical standard. Prior to the investigation, the three samples of each type were merged and cut into smaller pieces to avoid closed porosity. Furthermore, absorbed water was minimized by applying a vacuum for 12 h and afterwards storing the samples in sealed falcon tubes. The weight of the samples was determined after the measurements to exclude the weight of adsorbed gases and moisture.

Using the skeletal density $\rho_{S}$ and the bulk density $\rho_{B}$, the porosity P was calculated as follows [69]:

(1) $$P\left(\%\right)=\left(1-\frac{\rho_{B}}{\rho_{S}}\right)$$

The specific surface area and pore sizes were determined by means of nitrogen sorption, using an Autosorb IQ of Quantachrome (3P instruments) at −195.85 °C, cooled with liquid nitrogen. The cut samples of the helium pycnometry measurements were measured. They were degassed and dried at 200 °C/12 h prior to the investigation. BET (Brunauer–Emmet–Teller equation) was used to evaluate the specific surface area. The BET pressure range was set using the Rouquerol-Plot to avoid uncertainties due to microporosity, following the recommendation of IUPAC [68]. The non-local density function theory (NLDFT) calculations were used to evaluate the pore sizes, exerting silica, cylindrical pore, and equilibrium calculation model. ASiQwin v 4.01 of Quantachrome Instruments was used for the evaluation of the nitrogen sorption measurements.

The specific pore volume $V_{P}$ (cm^3^g^−1^), as well as the mean pore diameter $D_{P}$, were calculated using $\rho_{B}$, $\rho_{S}$, and the specific surface area $S_{BET}$ as follows [69]:

(2) $$V_{P}=\frac{1}{\rho_{B}}-\frac{1}{\rho_{S}},$$

(3) $$D_{P}=\frac{4V_{P}}{S_{BET}}$$

After the nitrogen sorption measurements, the chemical environment of the samples was evaluated by solid-state nuclear magnetic resonance (NMR) spectroscopy, using an Avance 400 MHz (Bruker) spectrometer. The ^29^Si spectrum was measured at 79.44 MHz, whereas ^13^C and ^1^H were obtained at 100.56 MHz and 399.88 MHz, respectively. Magic angle spinning (MAS) was performed at 10 kHz with a 4 mm MAS HX double resonance probe. Pulse length (π/2) was set to 4.6 µs for ^1^H. For the ^13^C{^1^H} and ^29^Si{^1^H} spectra, cross polarization MAS contact times were set to 2 ms and the recycle delay was set to 2 s. A secondary reference of adamantane for ^1^H and ^13^C and tetrakis(trimethylsilyl)silane for ^29^Si was used. The peak area was estimated by fitting a Gaussian peak using Python’s scipy library [70].

After the nitrogen sorption measurements, attenuated total reflection Fourier-Transform Infrared Spectroscopy (ATR-FTIR) was measured with a Vertex 70 (Bruker Optik GmbH & Co. KG, Ettlingen, Germany) to determine the composition of the surface modification by the different silylation agents.

To determine the amount of carbon, hydrogen, and nitrogen inside the ground samples, elemental analysis was performed using a Thermo FlashEA 1112 Organic Elemental Analyzer (Thermo Fisher Scientific, Waltham, MA, USA). The standard deviation was calculated from two measurements and thereafter rounded to two digits. Likewise, the error propagation of the deduction of ethoxy and silyl groups was rounded up.

Thermogravimetry analysis (Netzsch STA409PC/PG) measurements were conducted up to 1000 °C under argon atmosphere at 15 °C per minute on the powders for the UN, the HM, and the TE samples and on monoliths of the same synthesis batch for the TM sample to assess the thermal stability. The TGA was coupled with mass spectrometry (Pfeiffer Omnistar) in Mass id mode, equipped with an SEM-detector, to analyze the gas stream.

Dried samples of another synthesis batch with an identical procedure were investigated by means of small-angle X-ray scattering measurements at Helmholtz Zentrum für Materialien und Energie (BESSY II Berlin, Germany) at the µSpot beamline of the Max Planck Institute of Colloids and Interfaces [71] to evaluate changes in the nanostructure, such as the size of primary particles. The setup and integration of the data by the directly programmable data analysis kit (DPDAK) [72] were reported elsewhere [37]. An in-house python script was used to estimate data uncertainty when normalizing over transmission, monitor (i.e., primary beam intensity), and glassy carbon (NIST SRM 3600) [73]. This reference was applied according to the instructions of the National Institute of Standards and Technology (NIST). The normalized data were evaluated by SasView v 5.0.5 (http://www.sasview.org/, accessed on 27 January 2023) to determine the size of primary particles, applying a “fractal” model, which was originally reported by Teixeira et al. [61]. As taken directly from the SasView User Documentation (https://www.sasview.org/docs/user/models/fractal.html, accessed on 27 January 2023), the following model was considered:

(4) $$I\left(Q\right)=\phi V_{\mathrm{block}}{\left(\rho_{\mathrm{block}}-\rho_{\mathrm{solvent}}\right)}^{2}P\left(Q\right)S\left(Q\right)+\mathrm{background}.$$

(5) $$P\left(Q\right)=F{\left(QR_{0}\right)}^{2}$$

(6) $$F\left(x=QR_{0}\right)=\frac{3\left(\sin x-x\cos x\right)}{x^{3}}\;.$$

(7) $$V_{\mathrm{block}}=\frac{4}{3}\;{\pi R}_{0}.$$

(8) $$S\left(Q\right)=1+\frac{D_{f}\;\Gamma \left(D_{f}-1\right)}{{\left[1+1/{\left(Q\xi \right)}^{2}\;\right]}^{\left(D_{f}-1\right)/2}}\frac{\sin\left[\left(D_{f}-1\right)\tan^{-1}\left(Q\xi \right)\right]}{{\left(QR_{0}\right)}^{D_{f}}}\;.$$

Here, the radially integrated scattered intensity I(Q) was a function of the volume fraction ϕ, the volume of a building block $V_{\mathrm{block}}$, the scattering length densities of the block and the solvent $\rho_{\mathrm{block}}$ and $\rho_{\mathrm{solvent}}$, as well as the form factor P(Q) and structure factor S(Q). Additional parameters of the structure factor were the radius of primary particles $R_{0}$, correlation length ξ, and the fractal dimension $D_{f}$. The background value was derived from the lowest intensity of the SAXS dataset; the volume fraction was taken from the porosity calculations. Air was considered as a solvent; thus, the scattering length density was set to zero, whereas the scattering length density of the solid was calculated using the measured skeletal densities and assuming a silica network. A constant lognormal polydisper sity (“PD ratio”) of the radius of primary particles was assumed. Since the higher Q values with their lower intensities were underestimated in the fit, the weighting was set to dI. The fitting range was cut off at the transition to the wide-angle X-ray scattering region. The following parameters were fitted: the scaling factor (“scale”), the radius of particles (“radius”), the fractal dimension (“fractal_dim”), and the cluster correlation length (“cor_length”). The restrictions of the radius, correlation length, and fractal dimension were taken from the literature [56]. Furthermore, we have previously shown the limits of the fractal dimension [37]. Additionally, the radius and correlation length were estimated roughly from the intercept between Porod and fractal region, or fractal and Guinier region as visualized in the literature [74]. The distribution of the radius was restricted between 0 and 1, assuming a lognormal function (80 “Npts”, 8 “Nsigs”). The restrictions, as well as the resulting fit parameters and fitting errors, can be seen in Tables S4 and S5.

Visualizations of the molecular structures, reactions, and calculations of the Connolly solvent excluded volume were created with ChemDraw (version 20.1.1) and Chem3D (version 20.1.1.125) by PerkinElmer.

## Figures and Tables

**Table 1:** Carbon and hydrogen content in the specimens studied in this work: unmodified UN, the hexamethyldisilazane-modified HM, the triethylchlorosilane-modified TE, and the trimethylchlorosilane-modified TM. The details are given in the text.

	Measured		Calculated *		Subtracted -OC_2_H_5_ **		Hydrogen Bonded in Adsorbed Water (H_2_O) or Silanol (-OH) End Groups ***	
					−0.09	−0.23		
	C [wt%]	H [wt%]	C [mol *]	H [mol *]	C [mol *]	H [mol *]	C [mol *]	H [mol *]
**UN**	1.09 ± 0.05	1.42 ± 0.02	0.09 ± 0.01	1.42 ± 0.02	/	1.19 ± 0.03	/	1.19 ± 0.04
**HM**	14.21 ± 0.03	3.65 ± 0.01	1.18 ± 0.01	3.65 ± 0.01	1.09 ± 0.01	3.42 ± 0.03	/	0.14 ± 0.03
**TE**	16.90 ± 0.04	3.70 ± 0.01	1.41 ± 0.01	3.70 ± 0.01	1.32 ± 0.01	3.47 ± 0.03	/	0.18 ± 0.03
**TM**	14.16 ± 0.20	3.44 ± 0.02	1.18 ± 0.02	3.44 ± 0.02	1.09 ± 0.02	3.21 ± 0.03	/	−0.05 ± 0.04

* amount of carbon and hydrogen (in mol in an arbitrary amount of 100 g). ** amount of carbon and hydrogen (in mol in an arbitrary amount of 100 g) after subtraction of ethoxy groups (-OC_2_H_5_) determined from the carbon content of the UN sample. *** amount of hydrogen (in mol in an arbitrary amount of 100 g) bonded in adsorbed water (H_2_O) or silanol (-OH) end groups after subtraction of respective functional groups, i.e., -C_2_H_5_ (TE sample) and -CH_3_ (HM and TM samples).

**Table 2:** Summary of the NLDFT maximum amount of pore diameter, specific cumulative pore volume, and specific surface area determined by nitrogen sorption measurements and NLDFT calculations, as well as the specific pore volume and mean pore width, as estimated from the bulk and skeletal densities for the unmodified UN, hexamethyldisilazane-modified HM, triethylchlorosilane-modified TE, and trimethylchlorosilane-modified TM samples.

	NLDFT Max. Amount of Pore Diameter [nm]	Specific Cumulative Pore Volume [cm^3^g^−1^]	Specific Surface Area [m^2^g^−1^]	Specific Pore Volume [cm^3^g^−1^]	Mean Pore Width [nm]
**UN**	6.3	0.7	767.5	0.83 ± 0.25	4.33 ± 1.01
**HM**	6.1	1.3	852.7	0.97 ± 0.06	4.55 ± 0.25
**TE**	7.0	1.5	930.5	1.15 ± 0.08	4.94 ± 0.32
**TM**	10.1	3.1	902.5	6.04 ± 0.16	26.77 ± 0.64

## Data Availability

The supporting information provides additional results and evaluations. Furthermore, the authors gladly provide data within commensurate scope.

## Supplementary Materials

Additional supporting information can be downloaded at: https://www.mdpi.com/article/10.3390/gels9020160/s1; Figure S1. Molecular structure (created with ChemDraw v20.1.1) of trimethylchlorosilane (TMCS), triethylchlorosilane (TECS), and hexamethyldisilazane (HMDS) reported for surface modification of ambient pressure dried silica aerogels; Figure S2. (A) ^13^C NMR prediction of -CH_3_ at 18.4 ppm and -CH_2_ at 58.9 ppm, and (B) ^1^H NMR estimation for peaks around 1.21 ppm and 3.83 ppm correlating with -CH_3_ and -CH_2_ of tetraethyl orthosilicate calculated by ChemDraw (v20.1.1); Figure S3. NMR estimation of hexamethyldisilazane for (A) the ^13^C NMR with a peak at 6.2 ppm correlating to -CH_3_ and (B) ^1^H NMR with a rough quality prediction for -NH resulting in one peak at 1.5 ppm and a good quality prediction for -CH_3_ at 0.08 ppm. The prediction of triethylchlorosilane for (C) the ^13^C NMR shows two peaks correlating to -CH_3_ at 4.8 ppm and -CH_2_ at 10.6 ppm, and (D) at 0.94 ppm and 0.67 ppm for the ^1^H NMR. The NMR estimation of trimethylchlorosilane for (E) the ^13^C NMR shows one peak at 6.8 ppm associated with -CH_3_, which was estimated for (F) the ^1^H NMR at 0.42 ppm. The NMR predictions were calculated by ChemDraw (v20.1.1); Table S1. ^29^Si NMR Peak evaluation of the unmodified UN, hexamethyldisilazane-modified HM, triethylchlorosilane-modified TE, and trimethylchlorosilane-modified TM samples with their respective Gaussian peak areas are shown. Negative heights were equated to being not applicable (N.A.); Table S2. ^13^C NMR Peak evaluation of the unmodified UN, hexamethyldisilazane-modified HM, triethylchlorosilane-modified TE, and trimethylchlorosilane-modified TM samples with their respective Gaussian peak areas are shown; Table S3. ^1^H NMR Peak evaluation of the unmodified UN, hexamethyldisilazane-modified HM, triethylchlorosilane-modified TE, and trimethylchlorosilane-modified TM samples with their respective Gaussian peak areas are shown. Negative heights were equated to being not applicable (N.A.); Table S4. Restrictions of the scale, radius of primary particles, fractal dimension (“fractal_dim”), and correlation length (“cor_length”) of the SasView evaluation that was set for the “fractal” model with a static background; Table S5. Parameters for the SasView evaluation of the unmodified UN, hexamethyldisilazane-modified HM, triethylchlorosilane-modified TE, and trimethylchlorosilane-modified TM samples. A “fractal” model with a static background and a lognormal radius polydispersity (PD ratio) distribution was applied. An asterisk (*) shows the fitted parameters. The scale, background, volume fraction (“volfraction”), radius of primary particles, polydispersity, fractal dimension (“fractal_dim”), correlation length (“cor_length”), scattering length density of the solid (“sld_block”) and the solvent (“sld_solvent”), as well as the fitting error (Chi squared) are shown; Figure S4. Small-angle X-ray scattering measurements and the calculated SasView models (red dashed line) of (A) the unmodified UN (blue), (B) hexamethyldisilazane-modified HM (orange), (C) triethylchlorosilane-modified TE (green) and (D) trimethylchlorosilane-modified TM (pink) samples; Figure S5. Photographs of dried the unmodified UN, hexamethyldisilazane-modified HM, triethylchlorosilane-modified TE and trimethylchlorosilane-modified TM sample of various synthesis batches and experiments; Figure S6. 2D slice with scale bar of the µCT measurements of the (A) unmodified UN sample, (B) hexamethyldisilazane-modified HM sample, (C) triethylchlorosilane-modified TE sample, and (D) trimethylchlorosilane-modified TM sample; Figure S7. Nitrogen sorption isotherms measurements of (A) the unmodified UN (blue), (B) hexamethyldisilazane-modified HM (orange), (C) triethylchlorosilane-modified TE (green) and (D) trimethylchlorosilane-modified TM (pink) sample are shown with their respective adsorption (Ads.) and desorption (Des.) branches; Figure S8. NL-DFT evaluations of the nitrogen sorption measurements of the unmodified UN (blue), hexamethyldisilazane-modified HM (orange), triethylchlorosilane-modified TE (green) and trimethylchlorosilane-modified TM (pink) sample for (A) pore width diameter distribution and (B) their total pore volume over the pore width diameter; Table S6. The silylation agent, the detected groups by FTIR, ratio of Q4, Q3, and Q2 and the groups detected by NMR, the TGA overall weight loss and the main gas species detected by MS are shown for the unmodified UN, hexamethyldisilazane-modified HM, triethylchlorosilane-modified TE, and trimethylchlorosilane-modified TM silica gels. The carbon and hydrogen content of the elemental analysis, as well as the residual hydrogen amount (after deduction of the ethoxy and silyl end groups) is shown. Furthermore, the sample volume, width and bulk density measured by µCT, skeletal density by helium pycnometry, and primary particle diameter by means of SAXS are summarized. The results of the nitrogen sorption measurements, with the maximum amount of pore diameter and cumulative pore volume determined by NLDFT, and the specific surface area by BET are shown. The results of the specific surface area, bulk, and skeletal density allowed us to calculate the porosity, specific pore volume, and mean pore width.
