# Supplementary material for: Origin of the Springback Effect in Ambient-Pressure-Dried Silica Aerogels: The Effect of Surface Silylation
Source: Gels. 2023 Feb 16;9(2):160. doi: 10.3390/gels9020160 (PMC9956377; doi:10.3390/gels9020160)
Supplement: Supplementary file 1 [file gels-09-00160-s001.zip › gels-2213818-supplementary.pdf]

gels MDPI

Supplementary information

Origin of the springback effect in ambient pressure dried silica aerogels: The effect of surface silylation

Fabian Zemke [1]*, Julien Gonthier [2], Ernesto Scoppola [2], Ulla Simon [1], Maged F. Bekheet [1], Wolfgang Wagermaier [2] and Aleksander Gurlo [1]

[1] Technische Universität Berlin, Faculty III Process Sciences, Institute of Materials Science and Technology, Chair of Advanced Ceramic Materials, Straße des 17. Juni 135, 10623 Berlin, Germany; www.tu.berlin/ceramics
[2] Max Planck Institute of Colloids and Interfaces, Department of Biomaterials, Am Mühlenberg 1, 14476 Potsdam, Germany; https://www.mpikg.mpg.de/biomaterials
* Correspondence: Fabian.zemke@ceramics.tu-berlin.de; Tel.: +49 (0)30 314-22653

Overview supplementary information:

- **Figure S1.** Molecular structure (created with ChemDraw v20.1.1) of trimethylchlorosilane (TMCS), triethylchlorosilane (TECS), and hexamethyldisilazane (HMDS) reported for surface modification of ambient pressure dried silica aerogels.
- **Figure S2.** A) ^{13C} NMR prediction of -CH3 at 18.4 ppm and -CH2 at 58.9 ppm, and B) ^{1H} NMR estimation for peaks around 1.21 ppm and 3.83 ppm correlating with -CH3 and -CH2 of tetraethyl orthosilicate were calculated by ChemDraw (v20.1.1).
- **Figure S3.** NMR estimation of hexamethyldisilazane for A) the ^{13C} NMR with a peak at 6.2 ppm correlating to -CH3 and B) ^{1H} NMR with a rough quality prediction for -NH resulting in one peak at 1.5 ppm and a good quality prediction for -CH3 at 0.08 ppm. The prediction of triethylchlorosilane for C) the ^{13C} NMR shows two peaks correlating to -CH3 at 4.8 ppm and -CH2 at 10.6 ppm, and D) at 0.94 ppm and 0.67 ppm for the ^{1H} NMR. The NMR estimation of trimethylchlorosilane for E) the ^{13C} NMR shows one peak at 6.8 ppm associated with -CH3, which was estimated for F) the ^{1H} NMR at 0.42 ppm. The NMR predictions were calculated by ChemDraw (v20.1.1).
- **Table S1.** ^{29Si} NMR Peak evaluation of the unmodified UN, hexamethyldisilazane-modified HM, triethylchlorosilane-modified TE, and trimethylchlorosilane-modified TM samples with their respective Gaussian peak areas are shown. Negative heights were equated to being not applicable (N.A.).
- **Table S2.** ^{13C} NMR Peak evaluation of the unmodified UN, hexamethyldisilazane-modified HM, triethylchlorosilane-modified TE, and trimethylchlorosilane-modified TM samples with their respective Gaussian peak areas are shown.
- **Table S3.** ^{1H} NMR Peak evaluation of the unmodified UN, hexamethyldisilazane-modified HM, triethylchlorosilane-modified TE, and trimethylchlorosilane-modified TM samples with their respective Gaussian peak areas are shown. Negative heights were equated to being not applicable (N.A.).
- **Table S4.** Restrictions of the scale, radius of primary particles, fractal dimension (“fractal_dim”), and correlation length (“cor_length”) of the SasView evaluation that was set for the “fractal” model with a static background.
- **Table S5.** Parameters for the SasView evaluation of the unmodified UN, hexamethyldisilazane-modified HM, triethylchlorosilane-modified TE, and trimethylchlorosilane-modified TM samples. A “fractal” model with a

static background and a lognormal radius polydispersity (PD ratio) distribution was applied. An asterisk (*) shows the fitted parameters. The scale, background, volume fraction ("volfraction"), radius of primary particles, polydispersity, fractal dimension ("fractal_dim"), correlation length ("cor_length"), scattering length density of the solid ("sld_block") and the solvent ("sld_solvent"), as well as the fitting error (Chi squared) are shown.

- **Figure S4.** Small-angle X-ray scattering measurements and the calculated SasView models (red dashed line) of A) the unmodified UN (blue), B) hexamethyldisilazane-modified HM (orange), C) triethylchlorosilane-modified TE (green) and D) trimethylchlorosilane-modified TM (pink) samples.
- **Figure S5.** Photographs of the dried unmodified UN, hexamethyldisilazane-modified HM, triethylchlorosilane-modified TE and trimethylchlorosilane-modified TM sample of various synthesis batches and experiments.
- **Figure S6.** 2D slice with scale bar of the µCT measurements of the A) unmodified UN sample, B) hexamethyldisilazane-modified HM sample, C) triethylchlorosilane-modified TE sample, and D) trimethylchlorosilane-modified TM sample.
- **Figure S7.** Nitrogen sorption isotherms measurements of A) the unmodified UN (blue), B) hexamethyldisilazane-modified HM (orange), C) triethylchlorosilane-modified TE (green) and D) trimethylchlorosilane-modified TM (pink) sample are shown with their respective adsorption (Ads.) and desorption (Des.) branches.
- **Figure S8.** NL-DFT evaluations of the nitrogen sorption measurements of the unmodified UN (blue), hexamethyldisilazane-modified HM (orange), triethylchlorosilane-modified TE (green) and trimethylchlorosilane-modified TM (pink) sample for A) the pore width diameter distribution and B) their total pore volume over the pore width diameter.
- **Table S6.** The silylation agent, the detected groups by FTIR, ratio of Q4, Q3, and Q2 and the groups detected by NMR, the TGA overall weight loss and the main gas species detected by MS are shown for the unmodified UN, hexamethyldisilazane-modified HM, triethylchlorosilane-modified TE, and trimethylchlorosilane-modified TM silica gels. The carbon and hydrogen content of the elemental analysis, as well as the residual hydrogen amount (after deduction of the ethoxy and silyl end groups) is shown. Furthermore, the sample volume, width and bulk density measured by µCT, skeletal density by helium pycnometry, and primary particle diameter by means of SAXS are summarized. The results of the nitrogen sorption measurements, with the maximum amount of pore diameter and cumulative pore volume determined by NLDFT, and the specific surface area by BET are shown. The results of the specific surface area, bulk and skeletal density allowed us to calculate the porosity, specific pore volume, and mean pore width.

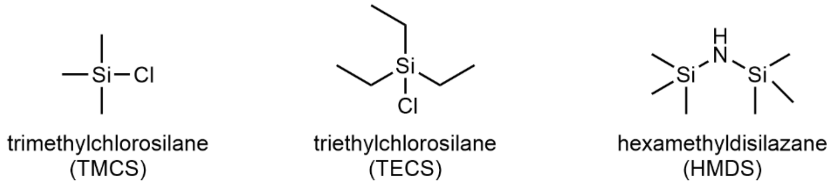

Figure S1. Molecular structure (created with ChemDraw v20.1.1) of trimethylchlorosilane (TMCS), triethylchlorosilane (TECS), and hexamethyldisilazane (HMDS) reported for surface modification of ambient pressure dried silica aerogels.

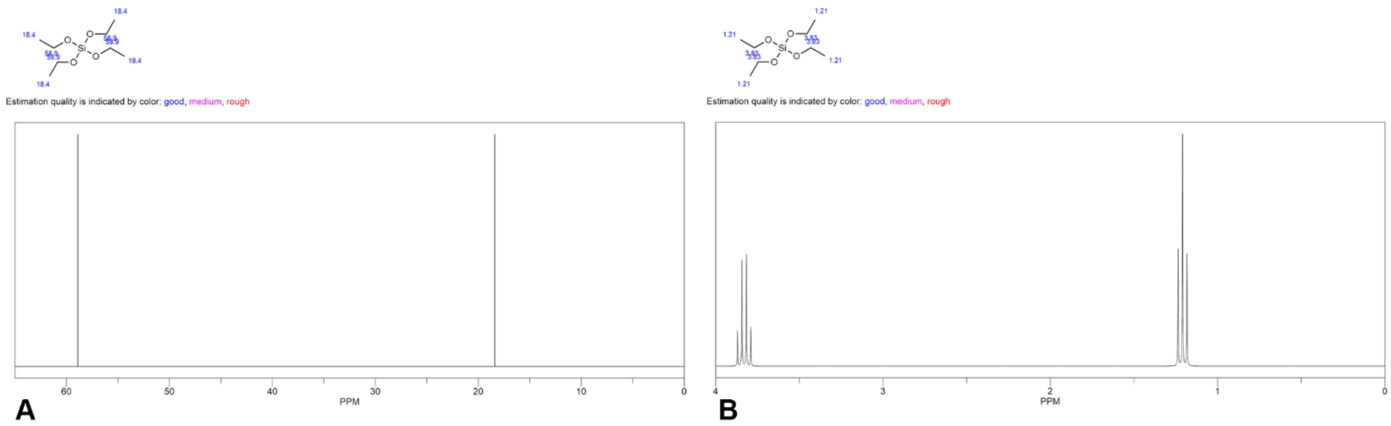

Figure S2. A) ^{13}C NMR prediction of $-CH_3$ at 18.4 ppm and $-CH_2$ at 58.9 ppm, and B) ^{1}H NMR estimation for peaks around 1.21 ppm and 3.83 ppm correlating with $-CH_3$ and $-CH_2$ of tetraethyl orthosilicate were calculated by ChemDraw (v20.1.1).

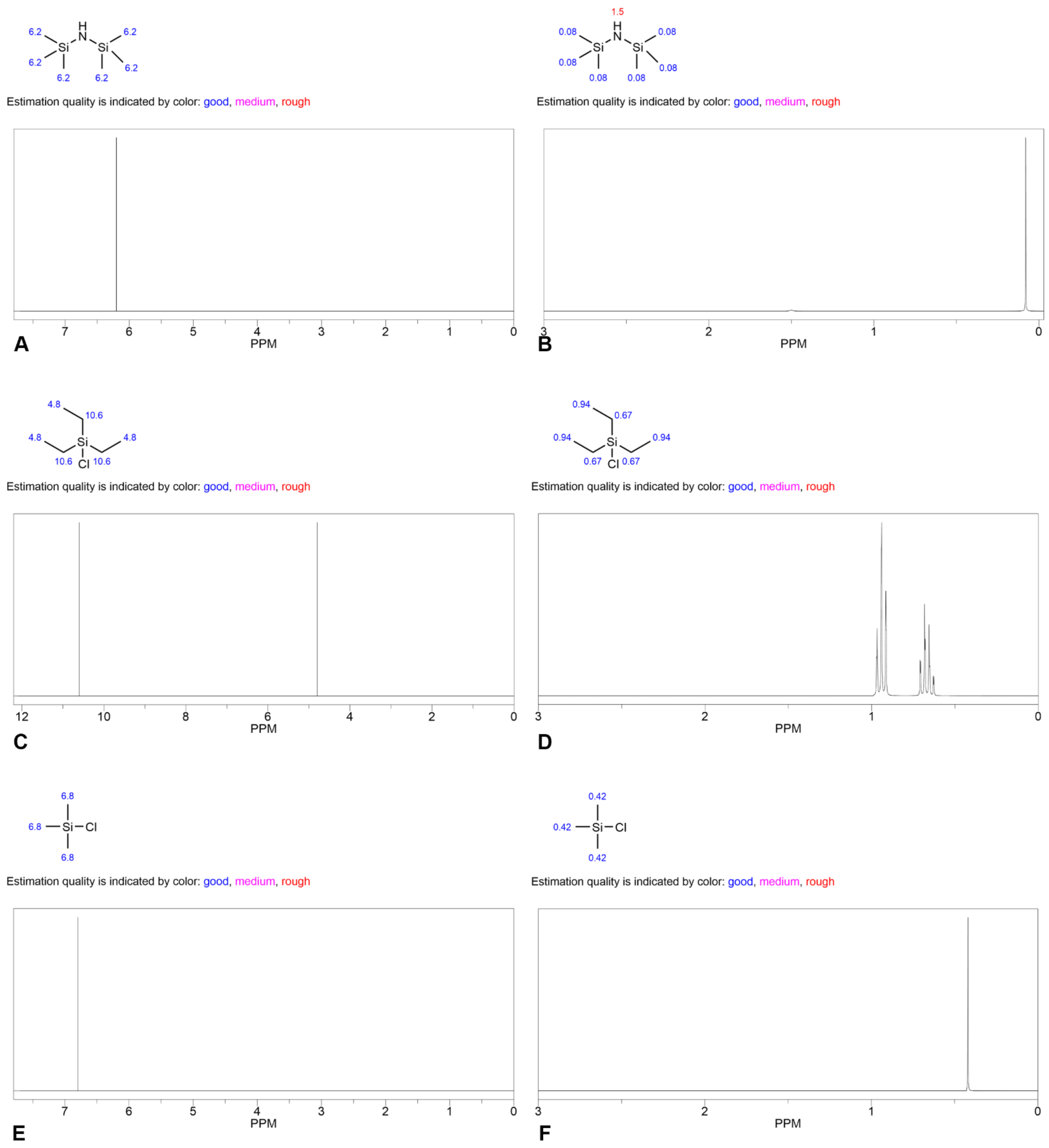

Figure S3. NMR estimation of hexamethyldisilazane for A) the ^{13}C NMR with a peak at 6.2 ppm correlating to $-CH_3$ and B) ^{1}H NMR with a rough quality prediction for -NH resulting in one peak at 1.5 ppm and a good quality prediction for $-CH_3$ at 0.08 ppm. The prediction of triethylchlorosilane for C) the ^{13}C NMR shows two peaks correlating to $-CH_3$ at 4.8 ppm and $-CH_2$ at 10.6 ppm, and D) at 0.94 ppm and 0.67 ppm for the ^{1}H NMR. The NMR estimation of trimethylchlorosilane for E) the ^{13}C NMR shows one peak at 6.8 ppm associated with $-CH_3$, which was estimated for F) the ^{1}H NMR at 0.42 ppm. The NMR predictions were calculated by ChemDraw (v20.1.1).

Table S1. ^{29}Si NMR Peak evaluation of the unmodified UN, hexamethyldisilazane-modified HM, triethylchlorosilane-modified TE, and trimethylchlorosilane-modified TM samples with their respective Gaussian peak areas are shown. Negative heights were equated to being not applicable (N.A.).

^{29}Si	Position [ppm]	Offset	Height [a.u.]	Width [ppm]	Area [a.u.]
UN	12.23	0	-1.75	10.00	N.A.
	-91.97	0	1825.85	2.12	9703.69
	-101.13	0	6016.10	2.71	40871.47
	-110.36	0	1009.11	3.17	8019.25
HM	12.44	0	1037.26	1.70	4420.51
	-95.87	0	64.98	5.33	868.25
	-100.81	0	514.66	2.52	3251.30
	-109.61	0	477.04	2.95	3527.87
TE	14.73	0	2718.85	1.36	9269.58
	-92.30	0	366.67	1.81	1663.76
	-101.13	0	3002.25	2.72	20471.60
	-110.17	0	1697.04	3.15	13401.04
TM	12.41	0	5796.60	1.42	20634.68
	-94.61	0	360.18	2.97	2681.71
	-101.38	0	2823.01	2.58	18258.63
	-110.19	0	3020.52	3.22	24382.24

Table S2. ^{13}C NMR Peak evaluation of the unmodified UN, hexamethyldisilazane-modified HM, triethylchlorosilane-modified TE, and trimethylchlorosilane-modified TM samples with their respective Gaussian peak areas are shown.

^{13}C	Position [ppm]	Offset	Height [a.u.]	Width [ppm]	Area [a.u.]
UN	58.42	0	1363.96	1.15	3938.72
	15.08	0	946.67	1.22	2904.21
	-2.26	0	353.67	1.07	951.98
HM	58.72	0	820.80	0.90	1853.48
	16.28	0	497.00	1.18	1474.52
	-0.66	0	19442.63	1.17	57017.04
TE	58.71	0	558.46	0.80	1122.69
	15.89	0	364.43	1.03	937.99
	4.64	0	6516.36	0.60	9813.30
TM	58.50	0	581.52	0.85	1242.16
	16.04	0	474.00	0.94	1118.55
	-1.14	0	3398.31	0.99	8414.60

Table S3. ^1H NMR Peak evaluation of the unmodified UN, hexamethyldisilazane-modified HM, triethylchlorosilane-modified TE, and trimethylchlorosilane-modified TM samples with their respective Gaussian peak areas are shown. Negative heights were equated to being not applicable (N.A.).

^1H	Position [ppm]	Offset	Height [a.u.]	Width [ppm]	Area [a.u.]
UN	4.53	0	64.24	1.32	213.15
	3.87	0	352.97	0.35	308.11
	3.59	0	-116.97	0.17	N.A.
	0.68	0	68.59	0.13	21.72
	-0.39	0	18.02	0.14	6.25
HM	3.14	8	30.08	0.32	24.08
	-0.37	0	639.89	0.38	613.41
TE	2.72	10	49.09	0.12	14.25
	0.47	0	263.80	0.12	80.75
	0.34	0	275.98	0.46	316.15
TM	3.35	9	15.00	0.23	8.49
	2.74	9	20.39	0.15	7.80
	0.68	0	71.65	0.35	62.06
	-0.39	0	339.88	0.31	266.51

Table S4. Restrictions of the scale, radius of primary particles, fractal dimension ("fractal_dim"), and correlation length ("cor_length") of the SasView evaluation that was set for the "fractal" model with a static background.

scale [/]		radius [Å]		fractal_dim [/]		cor_length [Å]	
min	max	min	max	min	max	min	max
0	inf	3	7	1.6	3.6	10	500

Table S5. Parameters for the SasView evaluation of the unmodified UN, hexamethyldisilazane-modified HM, triethylchlorosilane-modified TE, and trimethylchlorosilane-modified TM samples. A "fractal" model with a static background and a lognormal radius polydispersity (PD ratio) distribution was applied. An asterisk (*) shows the fitted parameters. The scale, background, volume fraction ("volfraction"), radius of primary particles, polydispersity, fractal dimension ("fractal_dim"), correlation length ("cor_length"), scattering length density of the solid ("sld_block") and the solvent ("sld_solvent"), as well as the fitting error (Chi squared) are shown.

	**scale* [/]**		**background [a.u.]**	**volfraction [/]**	**radius* [Å]**		**PD ratio lognormal [/]**	**fractal_dim* [/]**		**cor_length* [Å]**		**sld_block [10-6/Å2]**	**sld_solvent [10-6/Å2]**	**Chi squared [/]**
	**value**	**error**	**value**	**value**	**value**	**error**	**value**	**value**	**error**	**value**	**error**	**value**	**value**	**value**
**UN**	0.8986	0.0006	0.1078	0.33	3.20	0.01	0.50	3.42	0.01	14.75	0.02	19.5	0.0	664.00
**HM**	1.0629	0.0007	0.0314	0.41	4.05	0.01	0.50	2.64	0.01	26.90	0.09	12.2	0.0	1040.50
**TE**	4.0872	0.0032	0.0770	0.37	3.54	0.01	0.50	2.71	0.01	20.47	0.08	12.2	0.0	909.06
**TM**	0.7031	0.0007	0.0065	0.09	4.91	0.01	0.50	2.57	0.01	108.25	0.93	13.0	0.0	249.86

*Fitted parameter

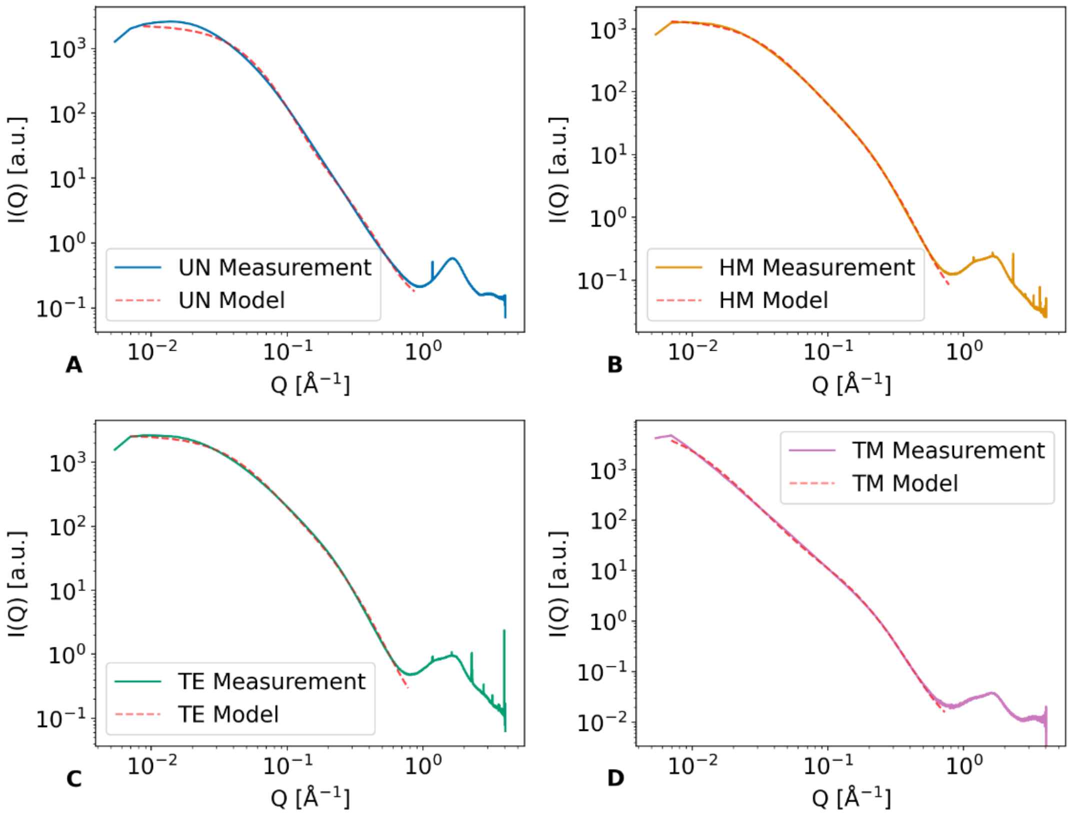

Figure S4. Small-angle X-ray scattering measurements and the calculated SasView models (red dashed line) of A) the unmodified UN (blue), B) hexamethyldisilazane-modified HM (orange), C) triethylchlorosilane-modified TE (green) and D) trimethylchlorosilane-modified TM (pink) samples.

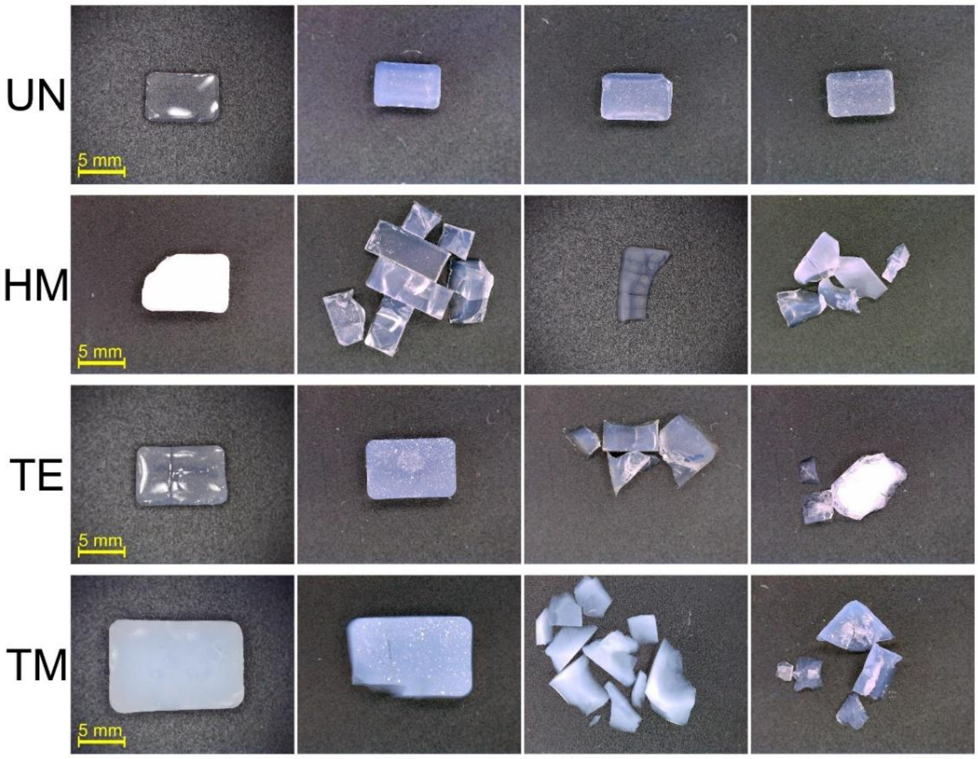

Figure S5. Photographs of the dried unmodified UN, hexamethyldisilazane-modified HM, triethylchlorosilane-modified TE and trimethylchlorosilane-modified TM sample of various synthesis batches and experiments.

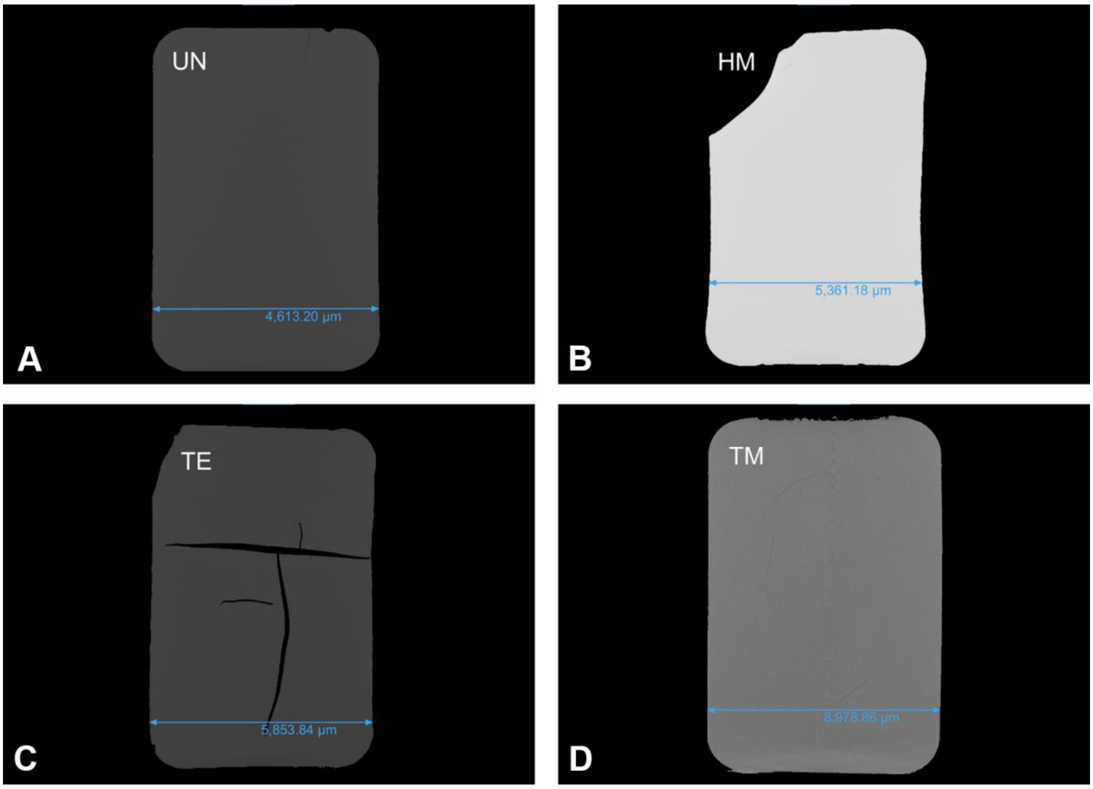

Figure S6. 2D slice with scale bar of the μCT measurements of the A) unmodified UN sample, B) hexamethyldisilazane-modified HM sample, C) triethylchlorosilane-modified TE sample, and D) trimethylchlorosilane-modified TM sample.

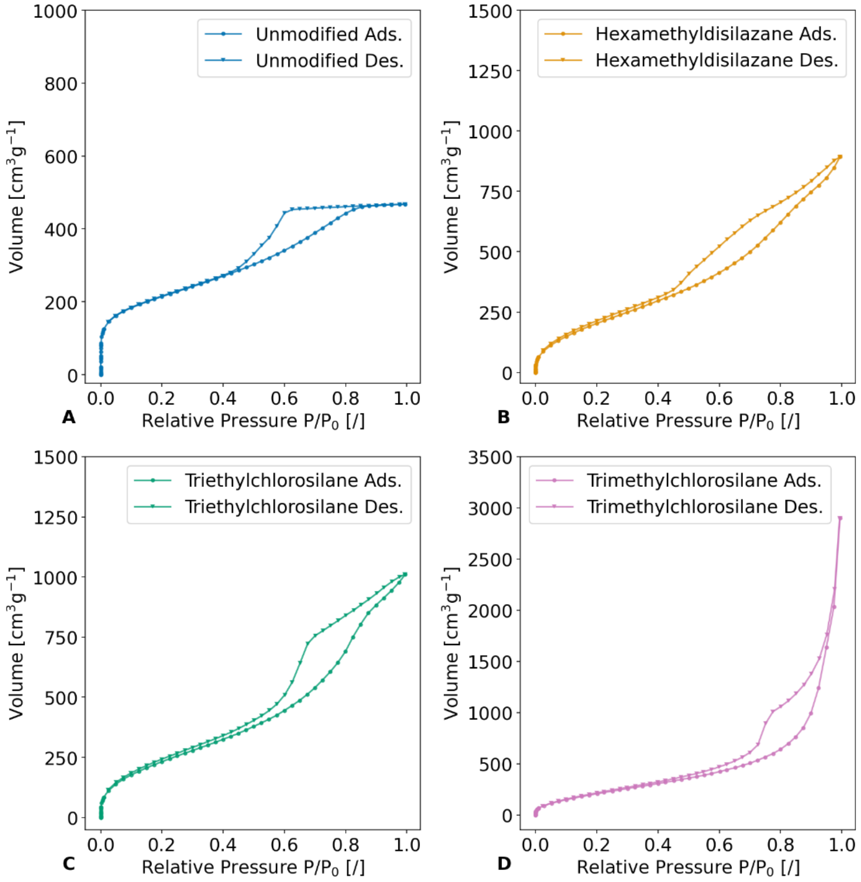

Figure S7. Nitrogen sorption isotherms measurements of A) the unmodified UN (blue), B) hexamethyldisilazane-modified HM (orange), C) triethylchlorosilane-modified TE (green) and D) trimethylchlorosilane-modified TM (pink) sample are shown with their respective adsorption (Ads.) and desorption (Des.) branches.

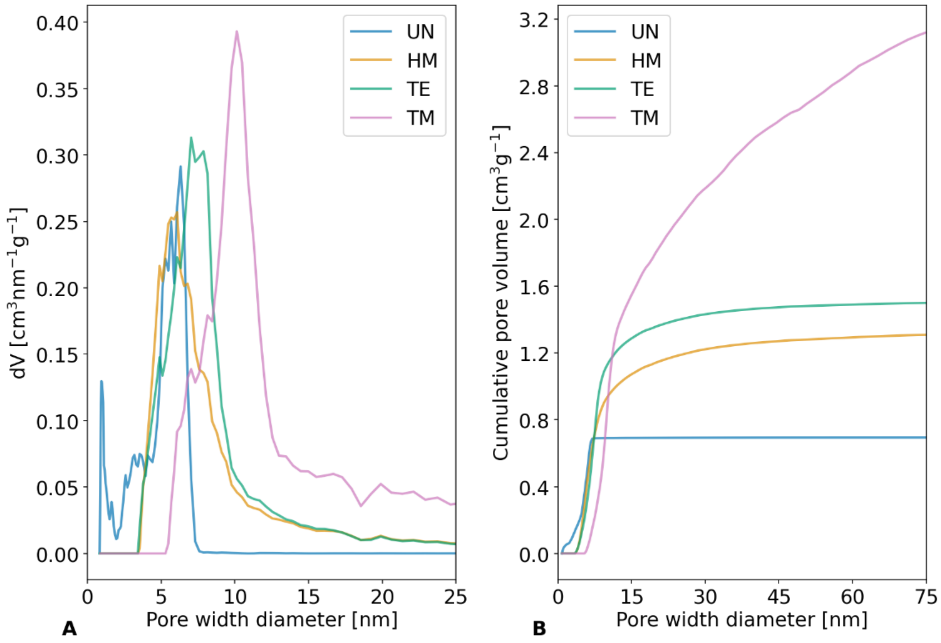

Figure S8. NL-DFT evaluations of the nitrogen sorption measurements of the unmodified UN (blue), hexamethyldisilazane-modified HM (orange), triethylchlorosilane-modified TE (green) and trimethylchlorosilane-modified TM (pink) sample for A) the pore width diameter distribution and B) their total pore volume over the pore width diameter.

Table S6. The silylation agent, the detected groups by FTIR, ratio of Q4, Q3, and Q2 and the groups detected by NMR, the TGA overall weight loss and the main gas species detected by MS are shown for the unmodified UN, hexamethyldisilazane-modified HM, triethylchlorosilane-modified TE, and trimethylchlorosilane-modified TM silica gels. The carbon and hydrogen content of the elemental analysis, as well as the residual hydrogen amount (after deduction of the ethoxy and silyl end groups) is shown. Furthermore, the sample volume, width and bulk density measured by μCT, skeletal density by helium pycnometry, and primary particle diameter by means of SAXS are summarized. The results of the nitrogen sorption measurements, with the maximum amount of pore diameter and cumulative pore volume determined by NLDFT, and the specific surface area by BET are shown. The results of the specific surface area, bulk and skeletal density allowed us to calculate the porosity, specific pore volume, and mean pore width.

		FTIR	*NMR*				*TGA*		*elemental analysis*						*μCT*			*Helium pycnometry*	*SAXS*	*Nitrogen sorption*			*calculated*				
	**Silylation agent**	**Main groups**	**Q4 [/]**	**Q3 [/]**	**Q2 [/]**	**Main groups**	**weight loss [%]**	**Main gas species**	**C [wt%]**		**H [wt%]**		**H [mol*]**		**Sample volume [mm^3]**	**Sample width [mm]**	**bulk density [$g\ cm^{-3}$]**	**skeletal density [$g\ cm^{-3}$]**	**Primary particle diameter [Å]**	**NLDFT pore diameter max. amount [nm]**	**Cumulative pore volume [cm^3g^{-1}]**	**Specific surface area [m^2g^{-1}]**	**Porosity [%]**	**Sp. Pore volume [cm^3g^{-1}]**		**Mean pore width [nm]**	
**UN**	None	Si-O-Si, OH, H_2O, SiOH	0.14	0.70	0.17	SiOH, H_2O, OCH_2CH_3, ($SiCH_3$)	10.51	H_2O, SiOH	1.09	±0.05	1.42	±0.02	1.19	±0.04	86.1	4.6	0.80	2.4	6.4	6.3	0.7	767.5	66.67	0.83	±0.25	4.33	±1.01
**HM**	HMDS	Si-O-Si, CH, SiC, OH, H_2O, SiOH	0.46	0.43	0.11	$SiCH_3$, OCH_2CH_3, (SiOH)	15.37	CH_3, C_2H_5	14.21	±0.03	3.65	±0.01	0.14	±0.03	140.5	5.4	0.61	1.5	8.1	6.1	1.3	852.7	59.33	0.97	±0.06	4.55	±0.25
**TE**	TECS	Si-O-Si, CH, SiC, OH, H_2O, SiOH	0.38	0.58	0.05	$SiCH_2CH_3$, OCH_2CH_3, (SiOH)	17.82	C_2H_5, CH_3	16.90	±0.04	3.70	±0.01	0.18	±0.03	173.0	5.9	0.55	1.5	7.1	7.0	1.5	930.5	63.33	1.15	±0.08	4.94	±0.32
**TM**	TMCS	Si-O-Si, CH, SiC, OH, (SiOH)	0.54	0.40	0.06	$SiCH_3$, OCH_2CH_3	10.41**	CH_3, C_2H_5	14.16	±0.20	3.44	±0.02	-0.05	±0.04	542.4	9.0	0.15	1.6	9.8	10.1	3.1	902.5	90.62	6.04	±0.16	26.77	±0.64

*Residual hydrogen amount of an arbitrary 100 g sample after deduction of the silanol and silyl groups.

**The TM sample was measured on a different sample geometry.
